# A hybrid framework: singular value decomposition and kernel ridge regression optimized using mathematical-based fine-tuning for enhancing river water level forecasting

**DOI:** 10.1038/s41598-025-90628-6

**Published:** 2025-03-04

**Authors:** Iman Ahmadianfar, Aitazaz Ahsan Farooque, Mumtaz Ali, Mehdi Jamei, Mozhdeh Jamei, Zaher Mundher Yaseen

**Affiliations:** 1 Department of Civil Engineering, Behbahan Khatam Alanbia University of Technology, Behbahan, Iran; 2 Canadian Centre for Climate Change and Adaptation, University of Prince Edward Island, St Peters Bay, PE Canada; 3 Faculty of Sustainable Design Engineering, University of Prince Edward Island, Charlottetown, PE Canada; 4 UniSQ College, University of Southern Queensland, Darling Heights, QLD 4305 Australia; 5 Department of Civil Engineering, Faculty of Civil Engineering and Architecture, Shahid Chamran University of Ahvaz, Ahvaz, Iran; 6 New Era and Development in Civil Engineering Research Group, Scientific Research Center, Al-Ayen University, Nasiriyah, Thi-Qar 64001 Iraq; 7 Khuzestan Water and Power Authority, Ahvaz, Iran; 8 Civil and Environmental Engineering Department, King Fahd University of Petroleum and Minerals, 31261 Dhahran, Saudi Arabia

**Keywords:** Water level forecasting, Singular value decomposition, Kernel ridge regression, Runge–Kutta algorithm, Light gradient boosting machine, Engineering, Mathematics and computing, Environmental sciences, Environmental chemistry, Environmental impact

## Abstract

The precise monitoring and timely alerting of river water levels represent critical measures aimed at safeguarding the well-being and assets of residents in river basins. Achieving this objective necessitates the development of highly accurate river water level forecasts. Hence, a novel hybrid model is provided, incorporating singular value decomposition (SVD) in conjunction with kernel-based ridge regression (SKRidge), multivariate variational mode decomposition (MVMD), and the light gradient boosting machine (LGBM) as a feature selection method, along with the Runge–Kutta optimization (RUN) algorithm for parameter optimization. The L-SKRidge model combines the advantages of both the SKRidge and ridge regression techniques, resulting in a more robust and accurate forecasting tool. By incorporating the linear relationship and regularization techniques of ridge regression with the flexibility and adaptability of the SKRidge algorithm, the L-SKRidge model is able to capture complex patterns in the data while also preventing overfitting. The L-SKRidge method is applied to forecast water levels in the Brook and Dunk Rivers in Canada for two distinct time horizons, specifically one- and three days ahead. Statistical criteria and data visualization tools indicates that the L-SKRidge model has superior efficiency in both the Brook (achieving R = 0.970 and RMSE = 0.051) and Dunk (with R = 0.958 and RMSE = 0.039) Rivers, surpassing the performance of other hybrid and standalone frameworks. The results show that the L-SKRidge method has an acceptable ability to provide accurate water level predictions. This capability can be of significant use to academics and policymakers as they develop innovative approaches for hydraulic control and advance sustainable water resource management.

## Introduction

Precise river water level predictions are imperative for flood alerts and efficient water resource management^1–3^. Researchers commonly use time series hydrological forecasting techniques to forecast future water level data. These forecasts provide significant insights into flood protection, catastrophe management, and resource management^2,4,5^. Forecasting river water levels (WL) is crucial for planning and addressing climate-related problems due to its dependence on hydro-meteorological aspects and inherent uncertainty. Precisely forecasting river flow patterns over high, medium, and low ranges is a challenging endeavor owing to their distinct nonlinear dynamics and hydrological processes. This complexity requires the creation of developed data-driven models^6–8^.

Diverse methodologies are employed to develop water level models that incorporate physical processes^9,10^. While physical models delve into the mechanism and dynamics of river flow, their adaptability is restricted by predictand variables, catchment conditions, and model parameters^11,12^. Nonlinearities arising from hydrological interactions and the mutual dependence of hydrological factors impede the predictive accuracy of physical models^13^. In contrast, data-driven methodologies aim to identify relationships among variables affecting water flow. They tackle the nonlinear attributes of predictor variables^14^. In conclusion, physical approaches use partial differential equations with constraints on boundaries, but data-driven models depend specifically on input-target parameter correlations, which simplifies model comprehending and reduces complexity.

The reliability of data-driven methods for water level prediction of rivers has been on the rise, as indicated by studies^15–17^. Employing artificial neural networks (ANN) as versatile tools, researchers have crafted single hidden layer of ANNs for forecasting both short-term^18,19^ and long-term^11^ variations in WL, treating the ANN as a black-box model. Wei created a forecasting methodology for river stages during typhoons in Taiwan’s Tanshui River Basin, comparing lazy (k-nearest neighbor (kNN) and locally weighted regression (LWR)) and eager (support vector regression (SVR), linear regression (REG), and artificial neural network (ANN)) learning approaches^20^. Using data from 50 typhoon events and hourly hydrological data from 1996 to 2007, results showed that ANN and SVR outperformed REG, while LWR was more effective than kNN, highlighting model-dependent performance. Deo and Şahin used the extreme learning machine (ELM) model to simulate average streamflow levels at three locations in eastern Queensland, showing its superiority compared to the ANN model^16^. The ELM model used nine predictors and ultimately resulted in acceptable precision measures (R^2^ = 0.964–0.997). Anh et al. developed a daily water level forecasting model based on the wavelet-ANN (WAANN) approach^21^. This method combined the benefits of wavelet method with ANN. Findings demonstrated that WAANN surpassed the conventional ANN model. Wang et al. devised a technique using a dilated causal convolutional neural network (DCCNN) which is capable of providing water-level predictions with lead durations ranging from 1 to 6 h^18^. The model was tested on a dataset of 16 typhoon events in Taiwan. The findings showed that the DCCNN outperformed existing support vector machine (SVM) and multilayer perceptron (MLP) methods.

Zhu et al. utilized the feed forward neural network (FFNN) and Deep learning (DL) to forecast monthly lake water levels in 69 temperate lakes in Poland for one month ahead^5^. The research indicated that the FFNN and DL models exhibited strong performance, with just negligible differences. The results showed that traditional methods might be enough to forecast lake water level. Phana and Nguyen introduced a hybrid method that included linear and nonlinear models^1^. Their methodology combines statistical MLMs with autoregressive integrated moving average (ARIMA) for predicting water levels.

To assess its efficacy, they applied this method to real datasets from the Vu Quang, Hanoi, and Hung Yen hydrological stations, revealing significant improvements in forecasting performance compared to other methods. Barzegar et al. improved long-term water level forecasts for Lake Michigan and Lake Ontario by combining the boundary corrected maximal overlap discrete wavelet transform (BC-MODWT) data preprocessing with a hybrid convolutional neural network (CNN) and long-short term memory (LSTM) (CNN-LSTM) model^22^. The suggested model’s performance was compared with the BC-MODWT-based MLMs, including random forest (RF) and SVR. From the results, the CNN-LSTM model performed better than the other models. An effective method to enhance the lake water level forecasting accuracy was shown using the BC-MODWT-CNN-LSTM model.

Masrur Ahmed et al. presented a novel hybrid DL model for the prediction of short-term river water levels (SWL)^15^. This model integrates the CNN, bi-directional long short-term memory (BiLSTM), and ant colony optimization (ACO) with a two-phase decomposition technique over several prediction time horizons. By combining variational mode decomposition (VMD), complete ensemble empirical mode decomposition with adaptive noise (CEEMDAN), and efficient feature extraction approaches, the CVMD-CBiLSTM model attained a high level of accuracy. Jamei et al. developed research aimed at forecasting daily floodwater levels in Australia’s Clarence River at the Baryulgil and Lilydale stations from 2005 to 2021^2^. Their novel methodology used a hybrid framework that integrated time-varying filter-based empirical mode decomposition (TVF-EMD) with feature selection using classification and regression trees (CART). Their results demonstrated that the CART-TVF-EMD-CFNN model surpassed other models at both stations.

Zakaria et al. utilized three machine learning algorithms (i.e., multi-layer perceptron neural network (MLP-NN), LSTM, and extreme gradient boosting (XGBoost)) to develop water level forecasting models for Malaysia’s Muda River^23^. The results showed that the MLP model had a higher accuracy of 0.871, which was superior to both the LSTM model (0.865) and the XGBoost model (0.831). Wang et al. evaluated five data preprocessing techniques VMD, wavelet packet decomposition (WPD), CEEMDAN, extreme-point symmetric mode decomposition (ESMD), and singular spectrum analysis (SSA)) in conjunction with a GRU model for forecasting monthly runoff^24^. The results showed that VMD and WPD increased accuracy. Xu and Wang^25^ presented an ensemble prediction model that employs least squares support vector machine (LSSVM) in conjunction with VMD, dung beetle optimization (DBO), and error correction (EC) to improve runoff forecast accuracy in the Ganjiang and Heihe River basins. The findings suggest that the proposed model could achieve high accuracy and enhance forecasts of extreme values.

Prior research has predominantly relied on the original version of MLMs and different types of optimization algorithms to derive optimal parameters^22,26^. The present study develops a novel version of Kernel Ridge regression (KRidge), called SKRidge, that combines the singular value decomposition (SVD) with Ridge regression. SKRidge enhances the precision and stability of the regression model by the integration of these two approaches. This makes it a potential method for predictive modeling. The proposed hybrid method integrates Ridge with SKRidge to create a linearly related model known as L-SKRidge. The Runge–Kutta (RUN) algorithm is used to improve control parameters for the L-SKRidge model in WL forecasting. Prior research has demonstrated the effectiveness and frequent usage of the RUN algorithm in various model tuning applications^27–29^. Therefore, the main goal of the present study is to evaluate the efficiency and capacity of MLMs in predicting water levels at various time frames in the future. The goal is to develop a thorough and dependable tool for academics and professionals that are interested in using MLMs for making water level projections in the future that extend beyond a single step. To the best of the authors’ awareness, the study introduces a pioneering attempt in crafting a novel structure for the kernel ridge regression model. It incorporates multivariate variational mode decomposition (MVMD) for real-world hydrological forecasting and employs the MARCOS methodology to identify the best machine learning model.

## Models background

The work aims to improve the accuracy and efficiency of regression analysis by using singular value decomposition (SVD) and Ridge regression to compute the regression parameters ($\psi$) for the KRidge model. SVD decomposes incoming data into orthogonal components, therefore separating salient characteristics. The regression variable ($\psi$) is derived from the SVD output. This enables the model to effectively utilize input variables. It captures complex relationships while maintaining stability against multicollinearity using Ridge regression’s L2 regularization.

To create the proposed model, L-SKRidge, it is systematically applied SVD to highlight essential features, computed the regression parameters ($\psi$), and refined them using Ridge regression. Eventually, L-SKRidge uses both strategies into a hybrid model that equilibrates the predictions of both the SKRidge and conventional Ridge procedures based on a linear combination. The suggested model is also optimized using the Runge–Kutta (RUN) method. This novel methodology seeks to significantly improve the precision and efficacy of regression analysis and provide a reliable instrument for forecasting.

To implement the proposed framework, several techniques are developed, including the proposed ML model, the optimization method, the measurement of alternatives and ranking according to compromise solution (MARCOS), the decomposition method, the feature selection approach, and the criterion evaluation. These methods are described at the following sections.

### linear regression

Multiple linear regression (MLR)^30^ outlines the association between the input variable *u* and a set of target variables {$x_{1}$, $x_{2}$, …, $x_{M}$}. A MLR with *M* independent variables can be represented as follows:

1 $$u = \phi_{0} + \phi_{1} x_{1} + \phi_{2} x_{2} + \cdots + \phi_{m} x_{m}$$

In Eq. (1), the training phase provides knowledge of the independent variables $x$ and dependent variables y, and the primary task is to determine the regression coefficients ($\phi$).

### Ridge regression

Multicollinearity issues can complicate MLR models, leading to challenges in accurate estimation. This complexity often leads to increased variance in the estimated regression coefficients and wider confidence intervals, resulting in significantly reduced estimation accuracy and poorer stability^31^. In simpler terms, in the MLR, when regression coefficients are substantial, even slight shifts in the data can have a substantial impact on prediction outcomes^31^.

Ridge regression is utilized to streamline the model by diminishing the significance of less important coefficients. It employs L_2_ regularization to the fitness function of linear regression, transforming the coefficient solution problem into a conditional optimization challenge^32^. This approach aims to simplify the coefficients and enhance the method’s capability. The fitness function of ridge regression is defined in Eq. (2).

2 $$Z\left( \phi \right) = \sum_{k = 1}^{K} \left( {u_{k} - \phi \cdot x_{ki} } \right)^{2} + \eta \left\| \phi \right\|_{2}^{2}$$

where $\eta$ has a positive amount. The $\eta$ parameter is used to regulate the $\left\| \phi \right\|_{2}^{2}$.

By derivative of Eq. (2), the parameter $\phi$ can be calculated by Eq. (3).

3 $$\phi = \left( {x^{T} x + \mu_{0} I} \right)^{ - 1} x^{T} u$$

where $I$ indicates the identity matrix, and $\mu_{0}$ expresses penalty coefficient. For computing the forecasted quantity, we establish the subsequent equation.

4 $$\hat{u}_{Ridge} = X\phi$$

Saunders and Gammerman^33^ advocated employing kernel functions (*Kr*) for customizing Ridge Regression (RR). The kernel ridge regression (KRidge) technique^33^ is used to derive Eq. (5), which builds upon the fundamental research of Vapnik^34^.

5 $$\hat{u} = h\left( x \right) = \mathop \sum \limits_{k = 1}^{K} \beta_{k} Kr\left( {x,x_{k} } \right)$$

The kernel function ($Kr\left( {x,x_{k} } \right)$) serves as a measure of relationship between variables, with the weights specified by $\beta_{k}$. The procedure for establishing these weights includes optimizing the quantity of modifications applied to the fitness function.

6 $$Z\left( \phi \right) = \sum_{k = 1}^{K} \left( {u_{k} - \phi \cdot x_{ki} } \right)^{2} + \eta \beta^{T} Kr\beta$$

Consequently, Eq. (7) resembles Ridge Eq. (3) with the sole difference being the substitution of all dot products with the *Kr* through the kernel method. To obtain an accurate value for the coefficients α, they can be defined by Eq. (7).

7 $$\beta = \left( {Kr + \mu I} \right)^{ - 1} X^{T} {\text{u}}$$

where $$U = \left( {U_{1} , \ldots ,U_{k} } \right)^{T}$$.

The KRidge employs the *Kr* as an input parameter to calculate the *Kr*. In this study, the matrix computation is accomplished through the utilization of an efficient wavelet function (WF). The proposed *Kr* can be formally formulated in Eq. (8)^35^.

8 $$Kr_{ij} = \cos \left( {\alpha \times \frac{{ - \left( {x_{i} - x_{j} } \right)^{2} }}{\delta }} \right) \times \exp \left( {\frac{{ - \left( {x_{i} - x_{j} } \right)^{2} }}{4 \times \rho }} \right)$$

where $\alpha$,$\delta$, and $\rho$ demonstrates three main unknown parameters of kernel function. In this work, the RUN optimization algorithm is employed to specify the main parameters of $Kr$.

### Singular value decomposition (SVD)

**Fig. 1:**
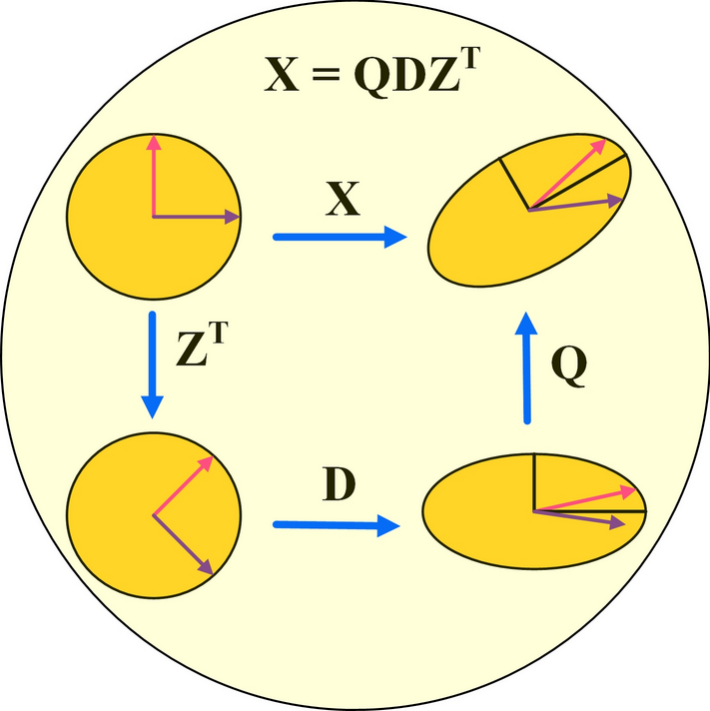
Conceptualization of the SVD.

The SVD^36^ is a commonly used method for analyzing an input by separating it into different components, which allows for the discovery of several important and interesting qualities that are intrinsic to the original input. The SVD has the capability to decrease the number of dimensions by means of matrix decomposition. Given any *i* × n matrix *X*, the SVD decomposition can be represented as follows:

9 $$X_{i \times n} = Q_{i \times i} {\text{D}}_{i \times n} Z_{n \times n}^{T} = QDZ^{T}$$

where $D$ is a matrix where the only non-zero elements are on the diagonal. These diagonal elements represent singular values of the matrix *X*. According to Fig. 1, in order to mathematically express *X*, three matrices must be applied sequentially: scaling (*D*), rotation by ($Z^{T}$), and further rotation by *Q*. Also, the *I* represents identity matrix and the *Q* and *Z* matrices reflect unit orthogonal vectors, it follows that:

10 $$Q^{T} Q = I,{ }Z^{T} Z = I$$

### Compute the regression variables employing the SVD and Ridge approaches.

In the present section, the SVD and Ridge approaches are used to calculate the regression parameters ($\psi$) for the KRidge model, which improves model precision. Here X can be substituted in Eq. (3) by its SVD derivation. Additionally, it will be performed calculations for ($X^{{\text{T}}} X$) and ($XX^{{\text{T}}}$):

11 $$X^{{\text{T}}} X = ZDQ^{T} QDZ^{{\text{T}}} = ZD^{2} Z^{T}$$

12 $$XX^{{\text{T}}} = QDZ^{T} ZDQ^{T} = QD^{2} Q^{{\text{T}}}$$

Applying Eqs. (11) and (12) in Eq. (3):

13 $$\psi = \left( {Z\left( {D^{2} + \mu_{0} I} \right)Z^{T} } \right)^{ - 1} ZDQ^{T} u$$

We can utilize the property that matrices H, J, and M adhere to the following relationship:

14 $$(HJM)^{ - 1} = {\text{M}}H^{ - 1} J^{ - 1}$$

15 $$\begin{aligned} \psi & = \underbrace {{\left( {Z^{T} } \right)^{ - 1} }}_{{\begin{array}{*{20}c} {\text{ Equals Z}} \\ {Z^{T} Z = I} \\ \end{array} }}\left( {D^{2} + \mu_{0} I} \right)^{ - 1} \underbrace {{Z^{ - 1} Z}}_{{\text{Equals I}}}DQ^{T} u \\ & = Z\left( {D^{2} + \mu_{0} I} \right)^{ - 1} DQ^{T} u \\ \end{aligned}$$

According to^37^, Eq. (3) can be resolved by employing SVD. Furthermore, matrix Q is identified as a lower-dimensional matrix.

### Hybrid SVD, KRidge, and ridge methods

This part provides a description of the proposed hybrid ML model to forecast the WL. In this regard, the KRdige uses the SVD to calculate the regression parameter ($\psi$). The SVD is applied to the input variables and allows the framework to generate more efficient inputs by simultaneously utilizing the properties of the Ridge and SVD approaches. Equation (16) is defined to construct the SKRidge.

16 $$\beta_{new} = \left( {Kr_{new} + \mu I} \right)^{ - 1} X^{T}_{new} { }u$$

in which

17 $$X^{T}_{new} = \psi X^{T}$$

18 $$Kr_{new} = Kr\left( {X_{new} ,X_{new,j} } \right)$$

Additionally, Eq. (19) is used to calculate the predicted variable ($\hat{u}$).

19 $$\hat{u}_{SKRR} = Kr_{new} \cdot \beta_{new}$$

**Fig. 2:**
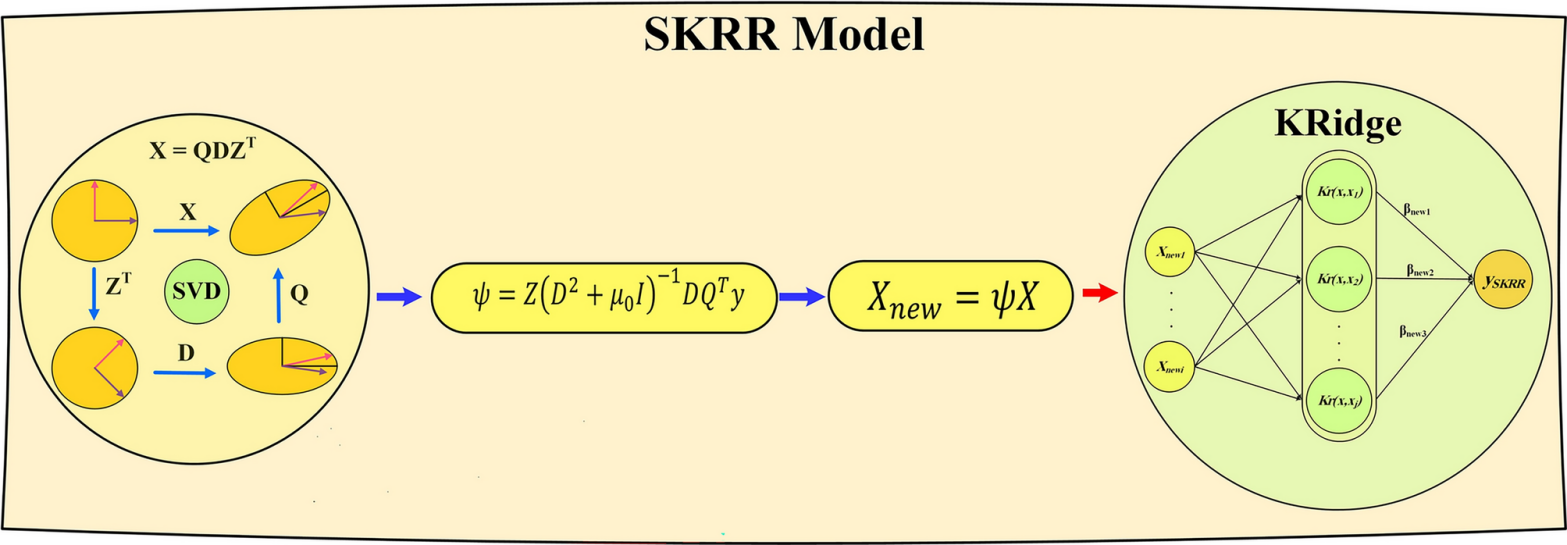
Schematic of the SKRidge model.

The objective of the present research was to improve the accuracy and efficiency of the framework. The proposed hybrid model (L-SKRidge) was developed by combining the SKRidge model with the Ridge approach through a linear connection. The hybrid model is shown as shown in Fig. 2. Pseudo code of the proposed model was presented in Algorithm 1.

20 $$\hat{u}_{L - SKRR} = \theta \cdot \hat{u}_{SKRR} + \left( {1 - \theta } \right) \cdot \hat{u}_{Ridge}$$

where $\theta$ has a positive amount within the range of [0, 1], and computed by the RUN algorithm.

**Algorithm 1:**
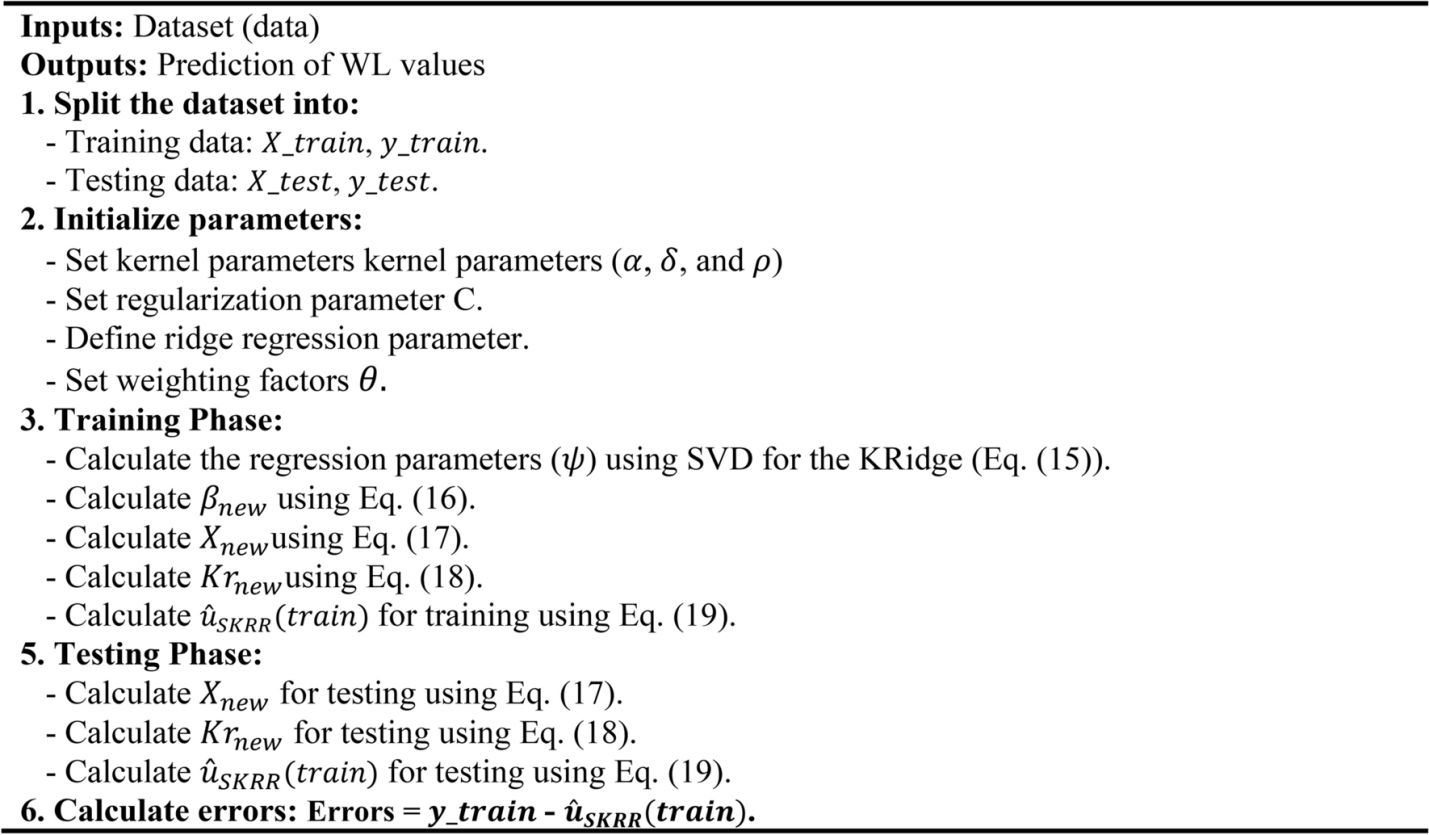
Pseudo-code of L-SKRidge method.

### RUN optimization method

Recently, researchers have developed numerous optimization algorithms to address various optimization problems. However, many of these methods draw inspiration from nature and lack a strong mathematical foundation^38,39^. Therefore, this study employs a mathematically grounded optimization technique known as Runge–Kutta optimization (RUN). The Runge–Kutta optimization algorithm was formulated with the foundation of the Runge–Kutta method^40^. The enhanced solution quality operator and the Runge–Kutta search mechanism (SM) make up the algorithm’s two operators. The RUN algorithm’s primary steps are described in the following subsections.

#### Updating solutions

The RUN algorithm is based on the RK method, and employs the SM to modify the current solution ($x_{k}$) at each iteration.

21 $$V_{1} = \left\{ {\begin{array}{*{20}c} {\left( {x_{rd1} + v \cdot A \cdot r \cdot x_{rd1} } \right) + A \cdot SM + \gamma \cdot randn \cdot {(}x_{rdb} - x_{rd} )} & {if rand < 0.5} \\ {\left( {x_{rdb} + v \cdot A \cdot r \cdot x_{rdb} } \right) + A \cdot SM + \gamma \cdot { }randn \cdot \left( {x_{c1} - x_{c2} } \right)} & {otherwise} \\ \end{array} } \right.$$

where $v$ is used to represent an integer with the value of either 1 or − 1; $r$ expresses a random coefficient in the range of [0, 2]; $x_{c1}$ and $x_{c2}$ express two positions, selected randomly within the range of [1, *N*]; *N* expresses the population size; $A$ indicates an adaptive coefficient; and $\gamma$ expresses a random coefficient. $SM$ is formulated as:

21-1 $$SM = \frac{1}{6}\left( {x_{RuKu} } \right)\Delta x$$

21-2 $$x_{RuKu} = w_{1} + 2 \times w_{2} + 2 \times w_{3} + w_{4}$$

21-3 $$w_{1} = \frac{1}{2\Delta x}\left( {rand \cdot x_{{\text{w}}} - \vartheta \cdot x_{{\text{b}}} } \right)$$

21-4 $$\vartheta = round\left( {1 + rand} \right) \cdot \left( {1 - rand} \right)$$

21-5 $$w_{2} = \frac{1}{2\Delta x}\left( {rand \cdot \left( {x_{{\text{w}}} + a_{1} \cdot k_{1} \cdot \Delta x} \right) - \left( {\vartheta \cdot x_{{\text{b}}} + a_{2} \cdot k_{1} \cdot \Delta x} \right)} \right)$$

21-6 $$w_{3} = \frac{1}{2\Delta x}\left( {rand \cdot \left( {x_{{\text{w}}} + a_{1} \cdot \left( {\frac{1}{2}w_{2} } \right) \cdot \Delta x} \right) - (\vartheta \cdot x_{{\text{b}}} + a_{2} \cdot \left( {\frac{1}{2}w_{2} } \right) \cdot \Delta x} \right)$$

21-7 $$w_{4} = \frac{1}{2\Delta x}\left( {r \cdot \left( {x_{{\text{w}}} + a_{1} \cdot w_{3} \cdot \Delta x} \right) - \left( {\vartheta \cdot x_{{\text{b}}} + a_{2} \cdot w_{3} \cdot \Delta x} \right)} \right)$$

where $x_{{\text{w}}}$ and $x_{{\text{b}}}$ indicate the worst and best positions; and $a_{1}$ and $a_{2}$ express two random numbers within the range of [0, 1]. $\Delta x$ is formulated as:

21-8 $$\Delta x = 2 \cdot rand \cdot \left| D \right|$$

21-9 $$D = rand \cdot \left( {\left( {x_{b} - rand \cdot x_{avg} } \right) + \chi } \right)$$

21-10 $$\chi = rand \cdot \left( {x_{k} - rand \cdot \left( {U - L} \right)} \right) \cdot {\text{exp}}\left( { - 4 \cdot \frac{It}{{MIt}}} \right)$$

where *D* indicates a random differential vector; $U$ and $L$ express the upper and lower bound; $It$ indicates the iteration count; $MIt$ indicates the upper limit for the number of iterations; and $x_{avg}$ indicates the average of solutions at each iteration. In this research, $x_{{\text{w}}}$ and $x_{{\text{b}}}$ express as follows:

21-11 $$\begin{gathered} {\varvec{if}} f\left( {x_{k} } \right) < f\left( {x_{bst,k} } \right) \hfill \\ \quad \quad x_{b} = x_{k} \hfill \\ \quad \quad x_{w} = x_{bst,k} \hfill \\ {\varvec{else}} \hfill \\ \quad \quad x_{b} = x_{bst,k} \hfill \\ \quad \quad x_{w} = x_{k} \hfill \\ {\varvec{end}} \hfill \\ \end{gathered}$$

where $x_{bst,k}$ represents the best solution that was achieved from three different solutions that were chosen randomly ($x_{c1}$, $x_{c2}$, and $x_{c3}$). $A$ is formulated as:

21-12 $$A = 2 \cdot {(0}{\text{.5}} - rand) \times F$$

21-13 $$F = 10 \times exp\left( { - 12 \cdot rand \cdot \left( {\frac{It}{{MIt}}} \right)} \right)$$

$x_{rdb}$ and $x_{rd1}$ are given by:

21-14 $$x_{rdb} = \omega \times x_{k} + \left( {1 - \omega } \right) \times x_{c1}$$

21-15 $$x_{rd1} = \omega \times x_{best} + \left( {1 - \omega } \right) \times x_{lbest}$$

where $\omega$ represents a number that has been independently generated and falls within the range of 0 to 1; $x_{best}$ indicates the best solution explored so far; and $x_{lbest}$ expresses the best position attained during the ongoing iteration.

#### Enhanced solution quality (ESQ)

RUN utilizes a robust operator termed the enhance solution quality (ESQ), denoting enhanced solution quality, to elevate solution quality and prevent the occurrence of local solutions. The RUN algorithm creates solution $V_{1}$ by employing the following method to carry out the ESQ operator.

22 $$\begin{gathered} {\varvec{if}} rand < 0.5 \hfill \\ \quad {\varvec{if}} w < 1 \hfill \\ \quad \quad \;V_{1} = x_{new} + \varphi \cdot \beta \cdot \left| {\left( {x_{new} - x_{avg} } \right) + randn} \right| \hfill \\ \quad {\varvec{else}} \hfill \\ \quad \quad \;V_{1} = \left( {x_{new} - x_{avg} } \right) + \varphi \cdot \beta \cdot \left| {\left( {2 \cdot rand \cdot x_{new} - x_{avg} } \right) + randn} \right| \hfill \\ \quad {\varvec{end}} \hfill \\ {\varvec{end}} \hfill \\ \end{gathered}$$

22-1 $$\beta = rand\left( {0{,} 2} \right) \cdot exp\left( { - \tau \cdot \left( {\frac{It}{{MIt}}} \right)} \right)$$

22-2 $$x_{avg} = \frac{{x_{c1} + x_{c2} + x_{c3} }}{3}$$

22-3 $$x_{new} = {\Omega } \times x_{avg} + \left( {1 - {\Omega }} \right) \times x_{best}$$

where ${\Omega }$ represents a random number that generates between 0 and 1. Additionally, $\tau$ signifies another random number, specifically 5 $\times rand$ value, while $\varphi$ represents an integer with possible values of 1, 0, or − 1.

As solution $V_{1}$ might not yield a superior objective function result compared to solution $x_{k}$ (i.e., where $f\left( {V_{1} } \right)> f(x_{k} )$), an additional attempt is made to generate a potential solution. This new solution, denoted as $x_{new3}$, is constructed through the following formulation:

23 $$\begin{gathered} {\mathbf{if}}\;{\varvec{rand}} < {\varrho } \hfill \\ \quad \quad \quad \quad \quad \quad V_{1} = \left( {V_{1} - rand \cdot V_{1} } \right) + AF \cdot \left( {rand \cdot x_{RuKu} + \left( {2 \cdot rand \cdot x_{b} - V_{1} } \right)} \right) \hfill \\ {\mathbf{end}} \hfill \\ \end{gathered}$$

in which

24 $$\varrho = rand\left( {0,2} \right) \times \exp \left( { - 5 \cdot rand \cdot \left( {\frac{It}{{MIt}}} \right)} \right)$$

**Fig. 3:**
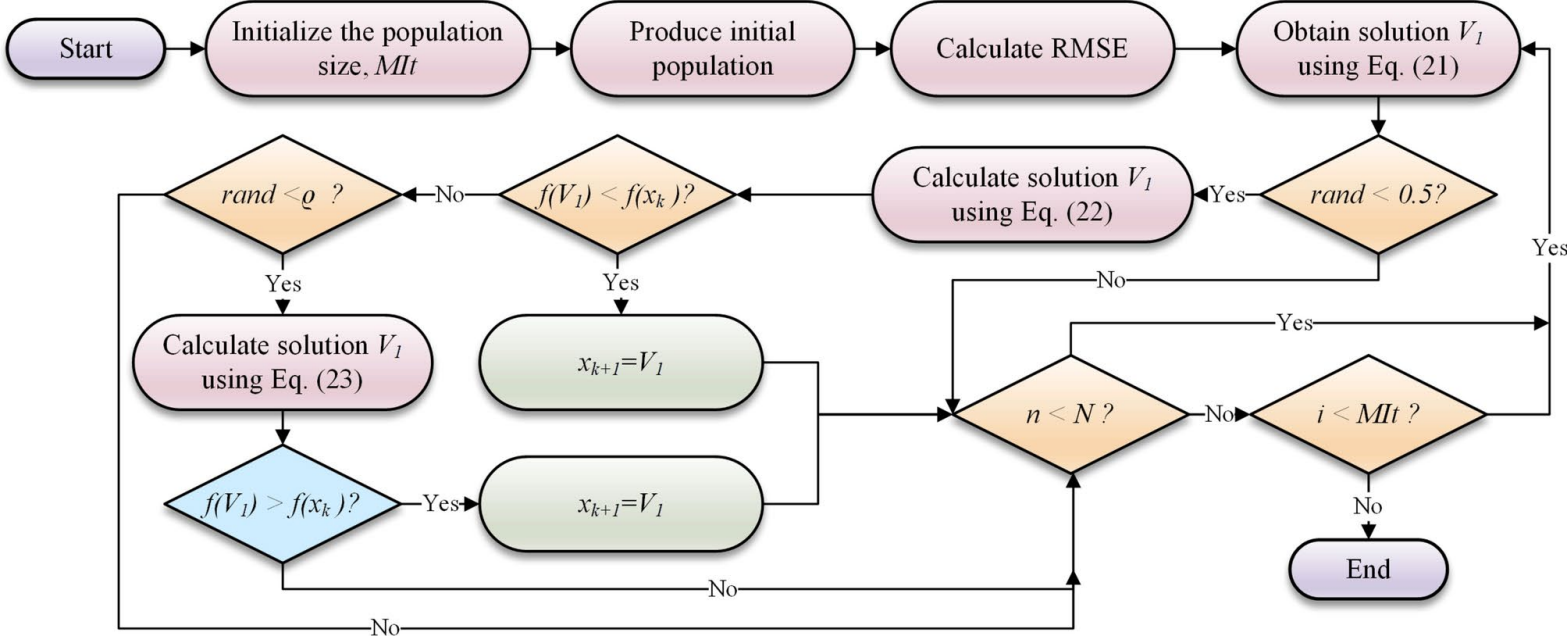
Flowchart of RUN algorithm.

In Eq. (23), $\varrho$ represents a dynamic parameter, and its value decreases over the last iteration. The Flowchart of RUN algorithm was depicted in Fig. 3.

### Measurement of alternatives and ranking according to compromise solution (MARCOS)

Analysis of many options in a given context is the focus of multi-criteria decision making (MCDM), a field of study with applications across many disciplines^41^. The MCDM is quickly becoming one of the most widely used decision-making techniques across many different fields. In this investigation, the problem was solved with the assistance of MARCOS due to its consistency and convenience of use with utility-based features in terms of both negative and positive solutions. The following is a description of the steps involved in the MARCOS method:

*Step 1*: Consider $P$ as the set of decision criteria ($P = \left\{ {P_{1} ,P_{2} , \ldots ,P_{j} } \right\}$) and *B* as the set of alternative routes ($B = \left\{ {B_{1} ,B_{2} , \ldots ,B_{l} } \right\}$). We begin by constructing an initial decision-making matrix. In this matrix, $AIS = \left\{ {x_{ais1} } \right.$, $\left. {x_{ais2} , \ldots ,x_{aisj} } \right\}$ represents the anti-ideal solution in the first row, while $IS = \left\{ {x_{is1} ,x_{{\text{is2 }}} , \ldots ,x_{{\text{isl }}} } \right\}$ represents the ideal solution in the last row. Therefore, the initial enlarged decision-making matrix is structured as outlined below:

25 $$Matrix = \left[ {\begin{array}{*{20}c} {x_{ais1} } & {x_{ais2} } & \ldots & {x_{aisl} } \\ {x_{11} } & {x_{12} } & \ldots & {x_{1l} } \\ {x_{21} } & {x_{22} } & \ldots & {x_{2l} } \\ \ldots & \ldots & \ldots & \ldots \\ {x_{j1} } & {x_{j2} } & \ldots & {x_{jl} } \\ {x_{is1} } & {x_{is2} } & \ldots & {x_{isl} } \\ \end{array} } \right]$$

where $AIS$ and $IS$ represent the least favorable and most favorable attributes for each decision criterion. Here, $x_{ij}$ corresponds to the performance of method *i* concerning decision criterion *j*. l denotes the number of methods, and j signifies the number of decision criteria, with *i* ranging from 1 to *l* and *k* ranging from 1 to *j*. The attribute values are computed using Eqs. (26) and (27)^42^.

26 $$AIS = \mathop {{\text{min}}}\limits_{i} x_{ik} {\text{ if }}j \in B,\mathop {{\text{max}}}\limits_{i} x_{ik} {\text{ if }}k \in nB$$

27 $$IS = \mathop {{\text{max}}}\limits_{i} x_{ik} {\text{ if }}j \in B,\mathop {{\text{min}}}\limits_{i} x_{ik} {\text{ if }}k \in nB$$

where *B* and *nB* pertain to the advantageous and disadvantageous decision criteria.

*Step 2*: The initial normalized decision matrix, denoted as $E =$ $\left[ {e_{ij} } \right]_{k \times j}$, is formed using the properties of decision criteria as defined in Eqs. (28) and (29):

28 $$e_{ij} = \frac{{x_{is} }}{{x_{ij} }}\;if\;k \in nB$$

29 $$e_{ij} = \frac{{x_{ij} }}{{x_{is} }}{\text{ if }}j \in B$$

where $e_{ij}$ denotes the normalized attribute of method $i$ and decision criterion $j$.

*Step 3*: The original weighted normalized decision matrix, denoted as $S = \left[ {s_{ij} } \right]_{k \times j}$, is created by multiplying the normalized characteristic with its associated weight as described in Eq. (30):

30 $$s_{ij} = e_{ij} \times w_{j}$$

where $w_{j}$ indicates the weighted normalized amount of method $i$ and decision criterion $j$.

*Step 4*: The utility degrees of AIS and IS are calculated utilizing Eqs. (31) and (32):

31 $$g_{i}^{ + } = \frac{{C_{i} }}{{C_{isi} }}$$

32 $$\begin{array}{*{20}r} \hfill {} & \hfill \\ \hfill {g_{i}^{ - } } & \hfill { = \frac{{C_{i} }}{{C_{aisi} }}} \\ \end{array}$$

where $C_{i} \left( {i = 1,2, \ldots ,j} \right)$ indicates the sum of the elements of matrix $s$, which is calculated by Eq. (33).

33 $$C_{i} = \mathop \sum \limits_{k = 1}^{j} C_{ik}$$

*Step 5*. Formulating the utility function (UF) for the evaluated option possibilities as specified in Eq. (34).

34 $$f\left( {g_{i} } \right) = \frac{{g_{i}^{ + } + g_{i}^{ - } }}{{1 + \frac{{1 - f\left( {g_{i}^{ + } } \right)}}{{f\left( {g_{i}^{ + } } \right)}} + \frac{{1 - f\left( {g_{i}^{ - } } \right)}}{{f\left( {g_{i}^{ - } } \right)}}}}$$

The UF related to the AIS is denoted as $f\left( {g_{i}^{ - } } \right)$, and the UF associated with the IS is expressed as $f\left( {g_{i}^{ + } } \right)$. These functions can be computed utilizing Eqs. (35) and (36) respectively.

35 $$f\left( {g_{i}^{ - } } \right) = \frac{{g_{i}^{ + } }}{{g_{i}^{ + } + g_{i}^{ - } }}$$

36 $$\begin{array}{*{20}r} \hfill {} & \hfill {} \\ \hfill {} & \hfill {f\left( {g_{i}^{ + } } \right) = \frac{{g_{i}^{ - } }}{{g_{i}^{ + } + g_{i}^{ - } }}} \\ \end{array}$$

where $f\left( {g_{i}^{ - } } \right)$ and $f\left( {g_{i}^{ + } } \right)$ represent the positive and negative UFs of method *i*.

### Decomposition method

To decompose the input dataset in this study, the MVMD method introduced by^43^ was used. The MVMD technique takes the standard variational mode decomposition (VMD) and expands it to a space with more than one dimension. The technique guarantees mode alignment across different variables and adds a useful feature for managing multivariate data^43^. In contrast to EMD, which use recursive filtering, MVMD makes advantage of the common frequency components that are present in multivariate datasets in order to produce intrinsic mode functions (IMFs). Below are the comprehensive stages of MVMD applied to input data x(t), composed of *N* channels, depicted as $\left[ {x_{1} \left( t \right),x_{2} \left( t \right), \cdots ,x_{N} \left( t \right)} \right]$:

1. Let’s consider the presence of M multivariate modulated fluctuations $\zeta_{l} \left( t \right)$ that satisfy the following assumption:
  37 $$x\left( t \right) = \mathop \sum \limits_{l = 1}^{M} \varrho_{l} \left( t \right)$$
  where $\varrho_{l} \left( t \right) = \left[ {\varrho_{l,1} \left( t \right),\varrho_{l,2} \left( t \right), \cdots ,\varrho_{l,N} \left( t \right)} \right]$, and $\varrho_{l,N} \left( t \right),l = 1,2, \cdots ,N$ represents the data’s $l$-th element corresponding to channel *n*.
2. To acquire the analytical representation $\varrho_{ + }^{l} \left( t \right)$ for each vector in $\varrho_{l} \left( t \right)$, the Hilbert transform is applied to calculate the one-sided frequency spectrum (OSFS). Subsequently, a complex exponential ${\text{e}}^{{ - j\psi_{l} t}}$ with a central frequency $\psi_{l} \left( t \right)$ is utilized to implement harmonic combination and shift the resultant OSFS to the baseband. The last step involves calculating the L_2_ norm of the gradient function of the harmonically conveyed $\varrho_{ + }^{l} \left( t \right)$ to provide an estimate of the bandwidth of each mode $\varrho_{l} \left( t \right)$.
  Because a singular frequency element (FE) $\psi_{l} \left( t \right)$ is used to execute harmonic combining of the full vector $\varrho_{ + }^{l} \left( t \right)$, it is essential to locate a common FE for multivariate fluctuations $\varrho_{l} \left( t \right)$ throughout multiple channels. This is because a single FE is used to carry out harmonic combining of the whole vector. Therefore, it is necessary for the collective influence of the IMFs to possess the capability to accurately decompose the original signal, while simultaneously minimizing the total bandwidth of the IMFs. Given the underlying assumption, the corresponding optimization problem with constraints could be expressed in the following manner^43^:
  38 $$\begin{array}{*{20}c} {\mathop {{\text{minmize}}}\limits_{{\left\{ {\varrho_{l,m} \left( t \right)} \right\},\left\{ {\psi_{l} } \right\}}} \left\{ {\mathop \sum \limits_{l} \mathop \sum \limits_{n} \left\| {\partial_{t} \left[ {\varrho_{ + }^{l,n} \left( t \right){\text{e}}^{{ - j\psi_{l} t}} } \right]_{2}^{2} } \right\|} \right\}} \\ {{\text{ subject to }}\mathop \sum \limits_{l} \varrho_{l,n} \left( t \right) = x_{n} \left( t \right),n = 1,2, \ldots ,N} \\ \end{array}$$
  where $\varrho_{ + }^{l,n} \left( t \right)$ indicates the analytical modulated signal associated with *l* in channel *n*; {$\varrho_{l,n} \left( t \right)$} represents a set of multivariate modulated oscillations; $\partial_{t} \left[ \cdot \right]$ denotes the partial derivative with respect to time; and {$\psi_{ln}$} indicates to a set of center frequencies of {$\varrho_{l,n} \left( t \right)$}.
3. To solve the variational problem that was described before, the additional Lagrange function is used as shown previously.
  39 $$\begin{aligned} L\left( {\left\{ {\varrho_{l,n} \left( t \right)} \right\},\left\{ {\psi_{l} } \right\},\sigma_{n} \left( t \right)} \right) & = \varphi \mathop \sum \limits_{l} \mathop \sum \limits_{n} \left\| {\partial_{t} \left[ {\varrho_{ + }^{l,n} \left( t \right){\text{e}}^{{ - j\psi_{l} t}} } \right]} \right\|_{2}^{2} + \mathop \sum \limits_{n} \left\| {x_{n} \left( t \right) - \mathop \sum \limits_{l} \varrho_{l,n} \left( t \right)} \right\| \\ & \quad + \mathop \sum \limits_{n} \left\langle {\sigma_{n} \left( t \right),x_{n} \left( t \right) - \mathop \sum \limits_{l} \varrho_{l,n} \left( t \right)} \right\rangle \\ \end{aligned}$$
  where $\varphi$ represents the penalty factor; $\sigma_{n} \left( t \right)$ indicates the Lagrange multiplier; $\left\langle { \cdot , \cdot } \right\rangle$ stands for the vectors’ inner product.
4. To continually update the variables $\varrho_{l,n} \left( t \right),\psi_{l}$ and $\sigma_{n} \left( t \right)$ in the frequency domain for a certain difficult unrestricted variational problem described in Eq. (40)^43^, the alternative direction technique of multipliers is utilized.
  40 $$\begin{gathered} \hat{\varrho }_{l,n}^{k + 1} \left( \psi \right) = \frac{{\hat{x}_{n} \left( \psi \right) - \mathop \sum \nolimits_{i \ne l} \hat{\varrho }_{l,n} \left( \psi \right) + \frac{{\hat{\sigma }_{c} \left( \psi \right)}}{2}}}{{1 + 2\theta \left( {\psi - \psi_{l} } \right)^{2} }} \hfill \\ \phi_{l}^{k + 1} \left( \psi \right) = \frac{{\mathop \sum \nolimits_{n} \mathop \smallint \nolimits_{0}^{\infty } \phi \left| {\hat{\varrho }_{l,n} \left( \psi \right)} \right|^{2} {\text{ d}}\psi }}{{\mathop \sum \nolimits_{n} \mathop \smallint \nolimits_{0}^{\infty } \left| {\hat{\varrho }_{l,n} \left( \psi \right)} \right|^{2} {\text{ d}}\psi }} \hfill \\ \hat{\sigma }_{n}^{k + 1} \left( \psi \right) = \hat{\sigma }_{n}^{k} \left( \psi \right) + \phi \left[ {\hat{x}_{n} \left( \psi \right) - \mathop \sum \limits_{l} \hat{u}_{l,n}^{k + 1} \left( \psi \right)} \right] \hfill \\ \end{gathered}$$
  where the signals in the frequency domain reflect the Fourier transform of the signals in the related time frame and are indicated by the notation $x_{n} \left( \psi \right)$ and $\sigma_{n} \left( \psi \right)$, respectively. The number of iterations is represented by the parameter *k*, whereas the time step is represented by the variable. By using the aforementioned update algorithms, it is feasible to dynamically divide the signal’s bandwidth, leading to the formation of *M* narrow-band IMFs. In addition, the MVMD algorithm has the ability to simultaneously handle numerous data channels. This functionality not only guarantees precise mode alignment across numerous channels but also enhances the resilience of the signal decomposition process.

### Feature selection

The existence of a large number of characteristics, which might make it difficult to recognize patterns, is a problem that often arises in the course of data analysis. As a result, one typical strategy is to make use of a feature selection technique with the end goal of reducing the total number of features^44,45^. In this regard, the light gradient boosting machine (LGBM) method is employed to specify the most important input variables. The LGBM is a highly efficient and scalable gradient boosting system that utilizes decision tree (DT) algorithms^46^. It is characterized by its quick execution, distributed computing capabilities, and superior efficiency. Many other machine learning applications use it for classification and feature selection. LGBM is an ensemble technique that combines the outputs of many decision trees (by a simple arithmetic addition) to generate a robust generalization^46,47^.

To develop an LGBM model with T trees, the iterative training process for a dataset of *n* data points can be described as follows^46^:

41 $$\hat{y}_{i}^{{\left( {{\text{i}}t} \right)}} = \mathop \sum \limits_{j = 1}^{n} h_{n} \left( {x_{j} } \right) = \hat{y}_{l}^{{\left( {{\text{i}}t - 1} \right)}} + h_{it} \left( {x_{j} } \right)$$

where $\hat{y}_{i}^{{\left( {{\text{i}}t} \right)}}$ indicates the estimation amount of the $j$-th at $it$-th iteration. The $h_{it}$ represents function for $it$-th DT.

The *hs* of each iteration can be determined by minimizing the following loss as much as feasible.

42 $$L^{{\left( {{\text{i}}t} \right)}} = \mathop \sum \limits_{n = 1}^{it} f\left( {y_{j} ,\hat{y}_{i}^{{\left( {{\text{i}}t} \right)}} } \right) + \mathop \sum \limits_{it = 1}^{Mit} {\upomega }\left( {h_{it} } \right)$$

The initial term represents the loss function between the actual and predicted values, while the regularization component is composed of ${\upomega }\left( {h_{it} } \right)$. LGBM stands as an implementation of gradient boosting decision trees (GBDT). LGBM uses two distinct strategies across the training and partitioning of each individual DT (h): gradient-based one-side sampling (GOSS) and leaf-wise growth. Consequently, the LGBM model is used to identify the best features for the WL forecasting in this study.

### Criterion evaluation

The ability of each MLM was assessed utilizing seven statistical metrics: R (correlation coefficient), Nash–Sutcliffe efficiency (NSE), RMSE (root-mean-square error), index of agreement (IA), U95% (uncertainty coefficient at a 95% confidence level), MAPE (mean absolute percentage error), and maximum absolute error (MaxAE)^28,48,49^. Statistical criteria were used to assess the accuracy and reliability of the models. The following equations outline the mathematical relationships for the criteria.

43 $$R = \frac{{\mathop \sum \nolimits_{k = 1}^{N} \left( {WL_{M,k} - \overline{WL}_{M} } \right) \times \left( {WL_{F,k} - \overline{WL}_{F} } \right) }}{{\sqrt {\mathop \sum \nolimits_{k = 1}^{N} (WL_{M,k} - \overline{WL}_{M} )^{2} \times \mathop \sum \nolimits_{k = 1}^{N} (WL_{F,k} - \overline{WL}_{F} )^{2} } { }}}$$

44 $$RMSE = \sqrt {\frac{1}{N} \mathop \sum \limits_{k = 1}^{N} (WL_{M,k} - WL_{F,k} )^{2} }$$

45 $$MaxAE = max_{k = 1, \ldots ,N} \left| {WL_{M,k} - WL_{F,k} } \right|$$

46 $$MAPE = \frac{1}{N} \mathop \sum \limits_{k = 1}^{N} \left| {\frac{{WL_{M,k} - WL_{F,k} }}{{WL_{M,k} }}} \right| \times 100$$

47 $$IA = 1 - \frac{{\mathop \sum \nolimits_{k = 1}^{N} \left( {WL_{F,k} - WL_{M,k} } \right)^{2} }}{{\mathop \sum \nolimits_{k = 1}^{K} \left( {\left| {\left( {WL_{F,k} - \overline{{WL_{F} }} } \right)} \right| + \left| {\left( {WL_{M,k} - \overline{{WL_{M} }} } \right)} \right|} \right)^{2} }}, 0 < IA \le 1$$

48 $$U_{95\% } = 1.96\sqrt {SD^{2} + RMSE^{2} }$$

49 $$NSE = 1 - \frac{{\mathop \sum \nolimits_{k = 1}^{N} \left( {WL_{M,k} - WL_{F,k} } \right)^{2} }}{{\mathop \sum \nolimits_{k = 1}^{N} \left( {WL_{M,k} - \overline{WL}_{M} } \right)^{2} }}$$

where the $WL_{F,k}$ and $WL_{M,k}$ indicate the forecasted and measured values of the WL, respectively. The averages of forecasted and measured values are determined by $\overline{WL}_{F}$ and $\overline{WL}_{M}$, respectively. *N* represents the size of the data collection, whereas SD represents the standard deviation. A suitable MLM would provide R and NSE values of 1, signifying perfect correlation and precision. Additionally, it would provide MAPE, RMSE, U_95%_, and MaxAE values of 0, indicating the best effectiveness.

## Modelling development

This research has developed a new modern intelligent framework for multi-temporal forecasting of the river water level in two case studies of Atlantic Canada province, Prince Edward Island (PEI). The aim of this research is to several hydro-meteorological signals like F, H, P, T, DP, and HD have been dedicated in the in Dunk and Brook Rivers over the 01/01/2015 to 31/12/2019. The MVMD decomposition constitutes the modern hybrid model, LGBM feature selection, and MCDM-based SVD-Kernel ridge regression scheme optimized by the RUN mathematical foundations algorithm to forecast the river WL. Also, to validate the primary model (MVMD-L-SKRidge), four advanced models, namely LASSO (Least Absolute Shrinkage and Selection Operator), CFNN, KRidge (Kernel Ridge Regression), and dRVFL (Deep random vector functional Link), were adopted in complementary (MVMD-KRidge, MVMD-dRVFL, MVMD-LASSO, and MVMD-CFNN) and standalone counterpart frameworks.

**Fig. 4:**
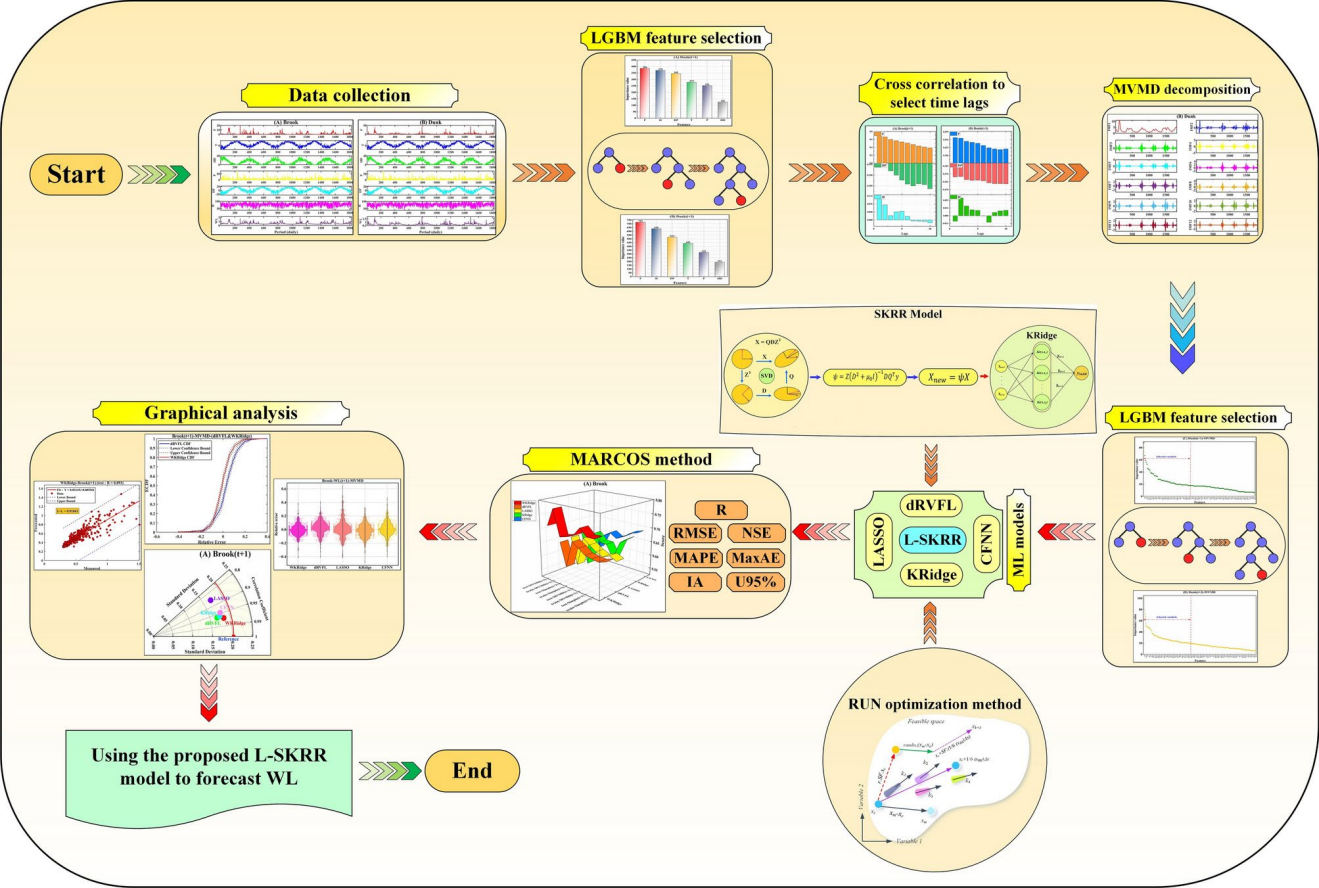
Graphical steps of WL forecasting using the proposed framework.

Here, the MVMD decomposition technique is performed in MATLAB programming tool. The rest of the model is developed in the Python platform using the MEALPY^50^, Scikit-learn^51^, and LGBM open-source libraries. All computational attempts have been performed on a laptop with the 3.10 GHz Intel Core i7 CPU and 8 GB RAM. All the modeling stages can be seen as a roadmap, as shown in Fig. 4. The main steps in the figure to forecast water level in Brook and Dunk Rivers are described as follows:

1. *Data collection*: The first step is to gather time series datasets t forecast water level in the Dunk and Brook rivers in Canada. In this regard, seven parameters (F, WL, T, HD, P, DP, and H) are gathered.
2. *Feature selection*: The LGBM method is used to select the most important features to predict water level fluctuations.
3. *Time lag selection*: Auto-Correlation Function (ACF), partial Auto-correlation function (PACF), and cross-correlation (CrC) analysis are employed to identify appropriate time lags.
4. *Decomposition of input variables*: Decomposition of input variables is carried out by the use of the MVMD technique. Various oscillatory patterns within the data can be discovered using the process of decomposing variables into IMFs.
5. *Feature importance in decomposed signals*: The LGBM method is once again used to identify the most significant IMFs. This stage guarantees the selection of the most important elements of the decomposing signals to decrease the dimension and more precise forecasts.
6. *Modeling and forecasting*: Five ML models (LASSO, CFNN, KRidge, dRVFL, and L-SKRidge) are implemented to forecast the water levels at both rivers.
7. *Model evaluation with MARCOS method*: The MARCOS method is applied to determine the best model. This method uses seven metrics (R, RMSE, MAPE, NSE, IA, MaxAE, and U_95%_) to specify the best model.
8. *Graphical analysis for model assessment*: Different graphical tools are used to evaluate and verify the ML models even further. These comprise scatter plots to compare model predictions against actual observations, violin plots to show relative error’s distribution and spread, Taylor plots to summarize model performance in pattern and amplitude terms, and empirical cumulative distribution function (ECDF) plots to evaluate the forecast error distribution among models.
9. *Specify and best model*: Graphical analysis and statistical metrics is used to select the best model for predicting water levels in the Brook and Dunk rivers. These evaluations ensure that the selected model not only predicts the actual dataset well, but also provides reliable and precise forecasts.

The main steps to prepare the input for the machine learning models are outlined as follows:

*Step 1*: Optimal estimation of the significant features for standalone models.

**Fig. 5:**
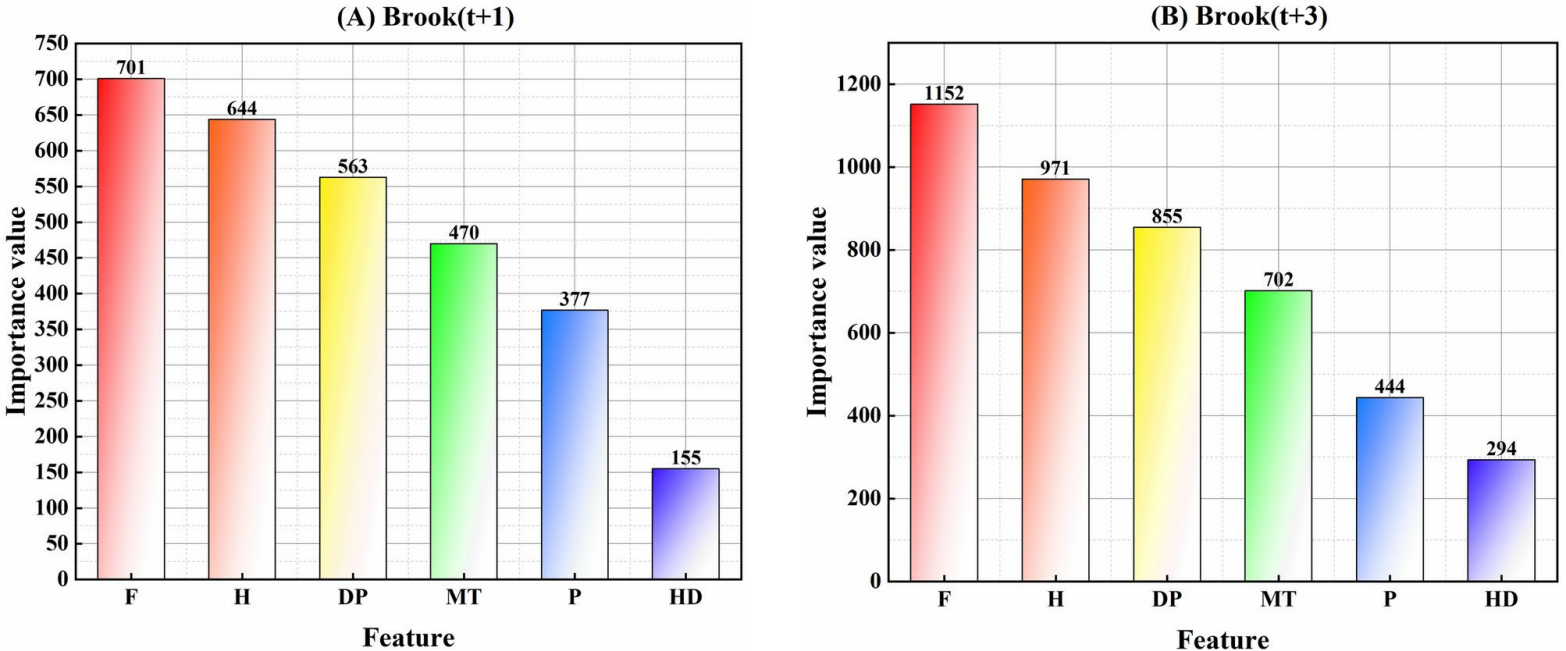
Significant feature extraction using the LGBM FS scheme for every horizon and case study.

Prior to applying any pre-processing procedures, the datasets are separated into 70% training and 30% testing subsets. This division is required to develop an artificial intelligence model capable of forecasting WL on a multi-temporal daily basis. In the first stage, all the meteorological factors were assessed to recognize the most important for every horizon and case study separately. Due to this, the LGBM feature (LGBM-FS) scheme has been implemented to evaluate all the features based on the importance values. Figure 5 and Appendix A depict the variation of the feature importance values of LGBM-FS for the Brook River. According to the results, it can be concluded that for all the underlying case studies, the F, H, and DP with the higher importance values have considered the influential feature to construct the forecasting models.

*Step 2*: Specify the time-lags.

**Fig. 6:**
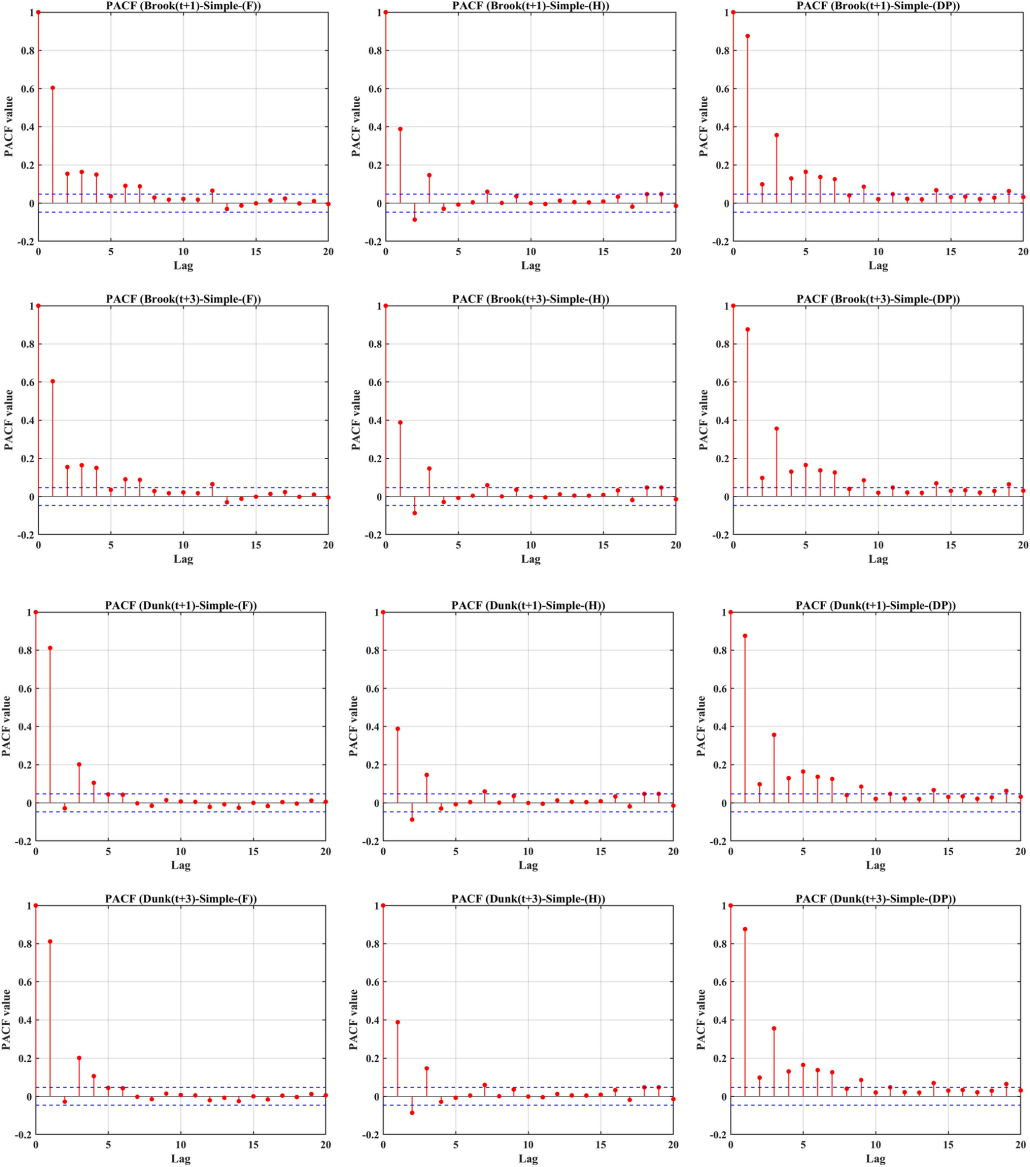
PACF examination of all the input features to construct the feeding components of the complementary models.

**Fig. 7:**
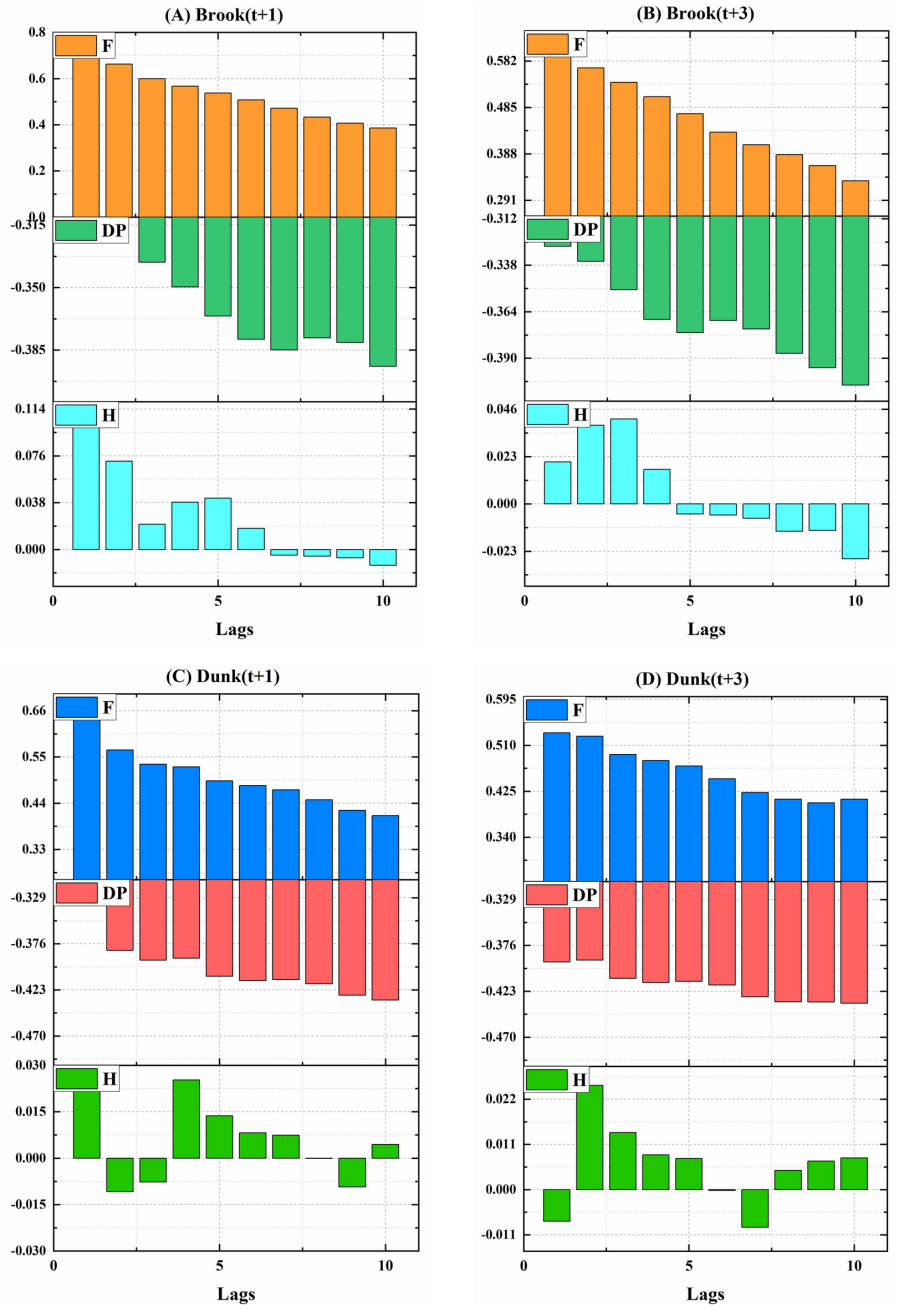
The outcomes of cross correlation to estimate the significant lags of the selected input features related to the simple models based on the importance values of the ten lags.

Given the highly non-stationary nature of the input signal, a robust technique is required to estimate the sub-sequence lags associated with optimal input features from the previous pre-processing stage. In this regard, in order to characterize the significant lags, sub-components of input signals for simple (standalone) models were extracted using ACF and the PACF associated with each selected input signal, including F, H, and DP in Brook and Dunk Rivers, for the -one and -three days ahead were computed for 20 lags to survey the best antecedent sub-components aimed at the hybrid model configuration. The results of ACF are displayed in Appendix B. In addition, the results of PACF are in Fig. 6. Based on the ACF, selecting the optimal time lags was challenging; therefore, the PACF was used. According to Fig. 6, the first three lags equivalent to the antecedent information associated with the previous three days were adopted as proper input features for reconstructing the simple (standalone models) in one and three days ahead horizons. In this research, to further confirm the selection of time lags, the cross-correlation (CrC) method was also applied (Fig. 7). Based on this figure and the PACF results, the first three lags were chosen to forecast the WL at the two stations.

**Fig. 8:**
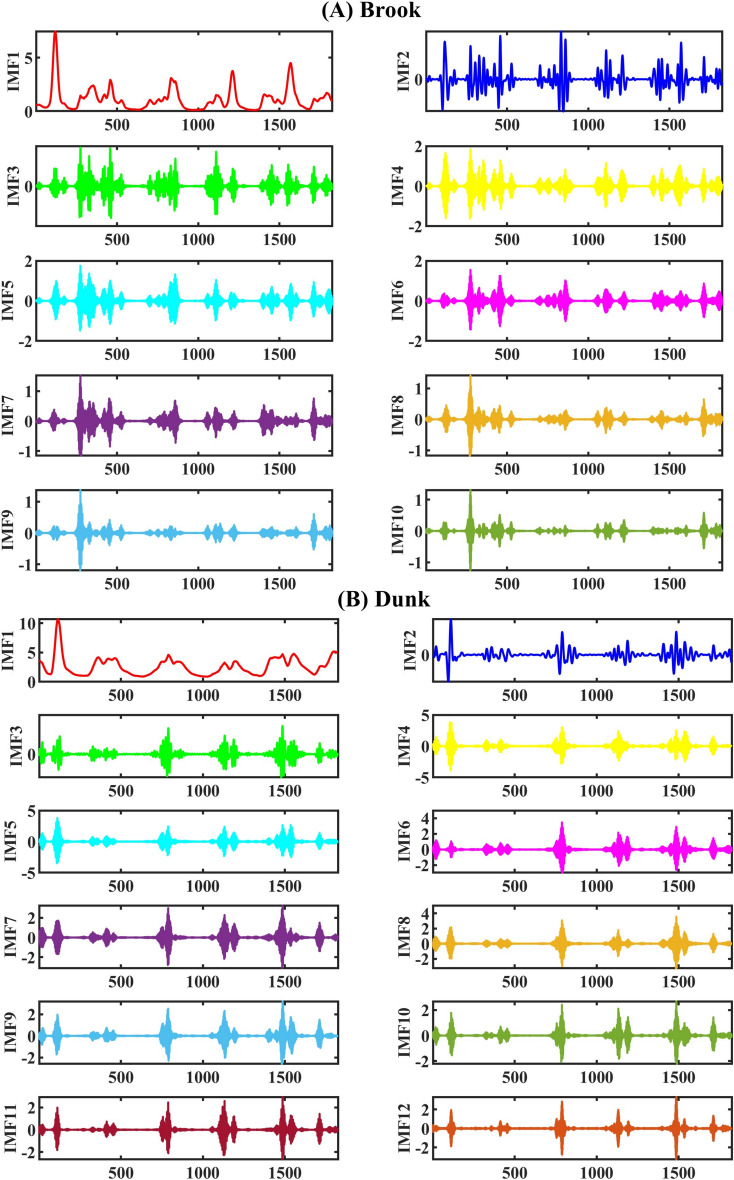
MVMD-based decomposition process of discharge signals as the sample in Boork (**A**) and Dunk (**B**) Rivers.

**Fig. 9:**
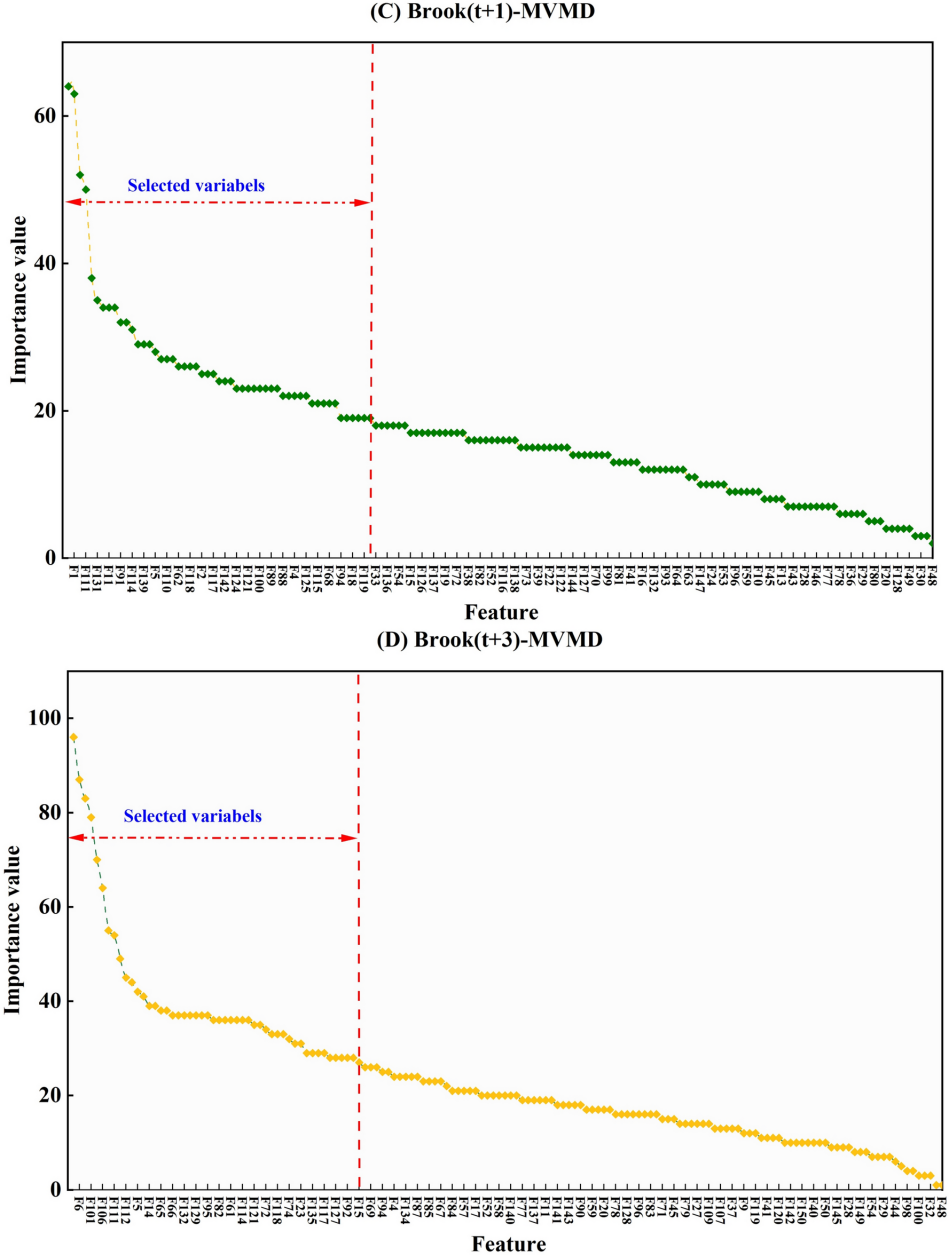
Outcomes of the LGBM-FS scheme on the most effective sub-components extraction among available pool of decomposed sub-sequences in every horizon for Brook river.

The MVMD approach decomposed all the input features to minimize signal complexity before putting them into the ML models. This was done to create the complimentary models. Figure 8 demonstrates the decomposed sub-components of the discharge flow parameter using the MVMD technique as a sample of the signal decomposition process. Here, the mode decomposition number (*k*) is the most important tuning parameter of the MVMD method, and it is a crucial component in achieving satisfactory precision. It is used a trial-and-error approach to determine the optimal value of k at each station. Following the aforementioned method, the best value of (*k*) for the Dunk River was 12 and for the Brook River it was 10. The default settings for the other setup parameters were used as a starting point: 2000 for Alfa, 10–7 for tolerance, zero for DC, and zero for Inti. The next step is to apply the three-lag time sub-sequences to the decomposed signals related to the features that were chosen. In order to improve the forecasting procedure’s precision and decrease computing costs, the most important components have been filtered through the LGBM-FS method. This process is executed with 30% of the whole dataset. The most promising decomposed features calculated using MVMD were selected from the pool to use in the ML models. Figure 9 reveals the results of the LGBM-FS technique for filtering the significant sub-components related to all the constructive features in the Brook and Dunk Rivers of PEI. It should be noted that the results of Dunk River are depicted in Appendix A.

*Step 3*: Machine learning configuration and tuning hyperparameters.

One of the most important phases of developing forecasting models is tuning ML model parameters. Using non-optimal hyperparameters could decrease the models’ precision and lead to an unfair comparison between the comparative prediction methods. For this purpose, the RUN method was used to optimize the main parameters (*θ*) of the L-SKRidge method. Notably, the main parameters of the other ML models (KRidge, LASSO, CFNN, and dRVFL) are optimized using the RUN algorithm. Tables 1 and 2 report the optimal values of the hyperparameter related to the ML models for Brook and Dunk Rivers, respectively. For instance, the main hyperparameters of the dRVFL and CFNN are the number of layers, neuron number, activation function, and hidden layer neuron number, respectively, their adjustments are more complicated than the KRidge and LASSO models.

**Table 1:** Adjust the control parameter quantities for simple-ML and MVMD-ML models to forecast the future behavior of Brook.

Time		Methods	Values of parameters
t + 1	Simple-ML	L-SKRR	$\alpha = 1.98E + 02$, $\delta = 2.04E + 01$, $\rho = 2.98E - 01$, $\mu = 1.71E - 03$, $\mu_{0} = 1.96E - 6$,$\theta = 0.71$
		KRidge	$\alpha = 3.59E + 09$, $\delta = 2.00E + 12$, $\rho = 5.17E + 08$,$\mu = 1.00E - 12$
		dRVFL	NoLs* = 10, NoNs = 20, Scf* = 200, Acf = sign, C = 1.00E+10
		LASSO	Alpha = 0.001
		CFNN	Structure = [5 3 1]
	MVMD-ML	L-SKRR	$\alpha = 1.62E + 10$, $\delta = 7.75E + 09$, $\rho = 2.14E + 09$, $\mu = 1.00E - 12$, $\mu_{0} = 1.00E - 3$,$\theta = 0.62$
		KRidge	$\alpha = 4.69E + 09$, $\delta = 2.00E + 12$, $\rho = 2.56E + 08$,$\mu = 4.63E - 14$
		dRVFL	NoLs = 20, NoNs = 300, Scf = 800, Acf = sign, C = 1.00E + 08
		LASSO	Alpha = 1.00E-08
		CFNN	Structure = [4 3 3 1]
t + 3	Simple-ML	L-SKRR	$\alpha = 5.30E + 00$, $\delta = 8.37E + 00$, $\rho = 1.29E - 02$, $\mu = 3.47E + 00$, $\mu_{0} = 9.73$,$\theta = 0.74$
		KRidge	$\alpha = 5.12E + 08$, $\delta = 3.49E + 11$, $\rho = 1.16E + 11$,$\mu = 1.00E - 12$
		dRVFL	NoLs = 5, NoNs = 50, Scf = 10, Acf = sign, C = 1.00E+10
		LASSO	Alpha = 1.00E−04
		CFNN	Structure = [5 5 1]
	MVMD-ML	L-SKRR	$\alpha = 1.04E + 04$, $\delta = 1.92E + 04$, $\rho = 1.91E + 03$, $\mu = 1.00E - 10$, $\mu_{0} = 0.78$,$\theta = 0.54$
		KRidge	$\alpha = 1.00E - 12$, $\delta = 1.49E + 10$, $\rho = 9.49E + 09$, $\mu = 1.00E - 10$,
		dRVFL	NoLs = 10, NoNs = 150, Scf = 100, Acf = sign, C = 1.00E+10
		LASSO	Alpha = 1.00E−10
		CFNN	Structure = [3 3 1]

NoNs* = Number of neurons, NoLs* = Number of layers, Acf * = activation function Scf* = scaling factor.

**Table 2:** Adjust the control parameter quantities for simple-ML and MVMD-ML models to forecast the future behavior of Dunk.

Time horizon		Models	Tuning parameter models
t + 1	Simple-ML	L-SKRR	$\alpha = 3.07E + 01$, $\delta = 1.42E + 02$, $\rho = 2.07E + 01$, $\mu = 1.63E - 07$, $\mu_{0} = 9.36$,$\theta = 0.65$
		KRidge	$\alpha = 6.66E + 10$, $\delta = 1.98E + 12$, $\rho = 1.73E + 12$,$\mu = 1.73E - 13$
		dRVFL	NoLs = 4, NoNs = 10, Scf = 200, Acf = sign, C = 2.00E+10
		LASSO	Alpha = 1.00E−10
		CFNN	Structure = [5 5 1]
	MVMD-ML	L-SKRR	$\alpha = 1.06E + 02$, $\delta = 3.16E + 01$, $\rho = 1.64E - 01$, $\mu = 1.12E - 03$, $\mu_{0} = 2.05E - 08$,$\theta = 0.82$
		KRidge	$\alpha = 6.66E + 10$, $\delta = 1.98E + 12$, $\rho = 1.73E + 12$,$\mu = 1.73E - 13$
		dRVFL	NoLs = 10, NoNs = 20, Scf = 200, Acf = sign, C = 2.00E+10
		LASSO	Alpha = 1.00E−06
		CFNN	Structure = [3 3 1]
t + 3	Simple-ML	L-SKRR	$\alpha = 1.98E + 01$, $\delta = 5.84E + 00$, $\rho = 2.00E + 01$, $\mu = 2.71E - 04$, $\mu_{0} = 1.81E - 05$,$\theta = 0.64$
		KRidge	$\alpha = 2.24E - 05$, $\delta = 7.99E + 02$, $\rho = 2.00E + 03$,$\mu = 7.99E - 02$
		dRVFL	NoLs = 5, NoNs = 20, Scf = 300, Acf = sign, C = 2.00E+10
		LASSO	Alpha = 1.00E−04
		CFNN	Structure = [4 3 1]
	MVMD-ML	L-SKRR	$\alpha = 1.00E - 20$, $\delta = 3.52E + 02$, $\rho = 2.19E - 02$, $\mu = 1.02E - 01$, $\mu_{0} = 1.45E - 03$,$\theta = 0.82$
		KRidge	$\alpha = 4.82E + 01$, $\delta = 1.98E + 03$, $\rho = 2.00E + 03$,$\mu = 2.47E - 02$
		dRVFL	NoLs = 5, NoNs = 100, Scf = 200, Acf = sign, C = 2.00E+10
		LASSO	Alpha = 1.00E−10
		CFNN	Structure = [3 3 1]

The datasets were normalized within the range of [0, 1] before they were utilized as inputs for the model’s goal and predictor variables. This normalization procedure guaranteed that each variable had a similar order of magnitude. A linear-based normalization technique was used to achieve this, which can be stated as follows^52^:

$$X_{N} = \frac{{X_{Real} - X_{MIN} }}{{X_{MAX} - X_{MIN} }}$$

where $X_{N}$ represents normalized input data, $X_{Real}$ represents real-time values data, $X_{MIN}$ represents the minimum real-time values, and $X_{MAX}$ denotes the maximum real-time values.

## Case study and data preparation

**Fig. 10:**
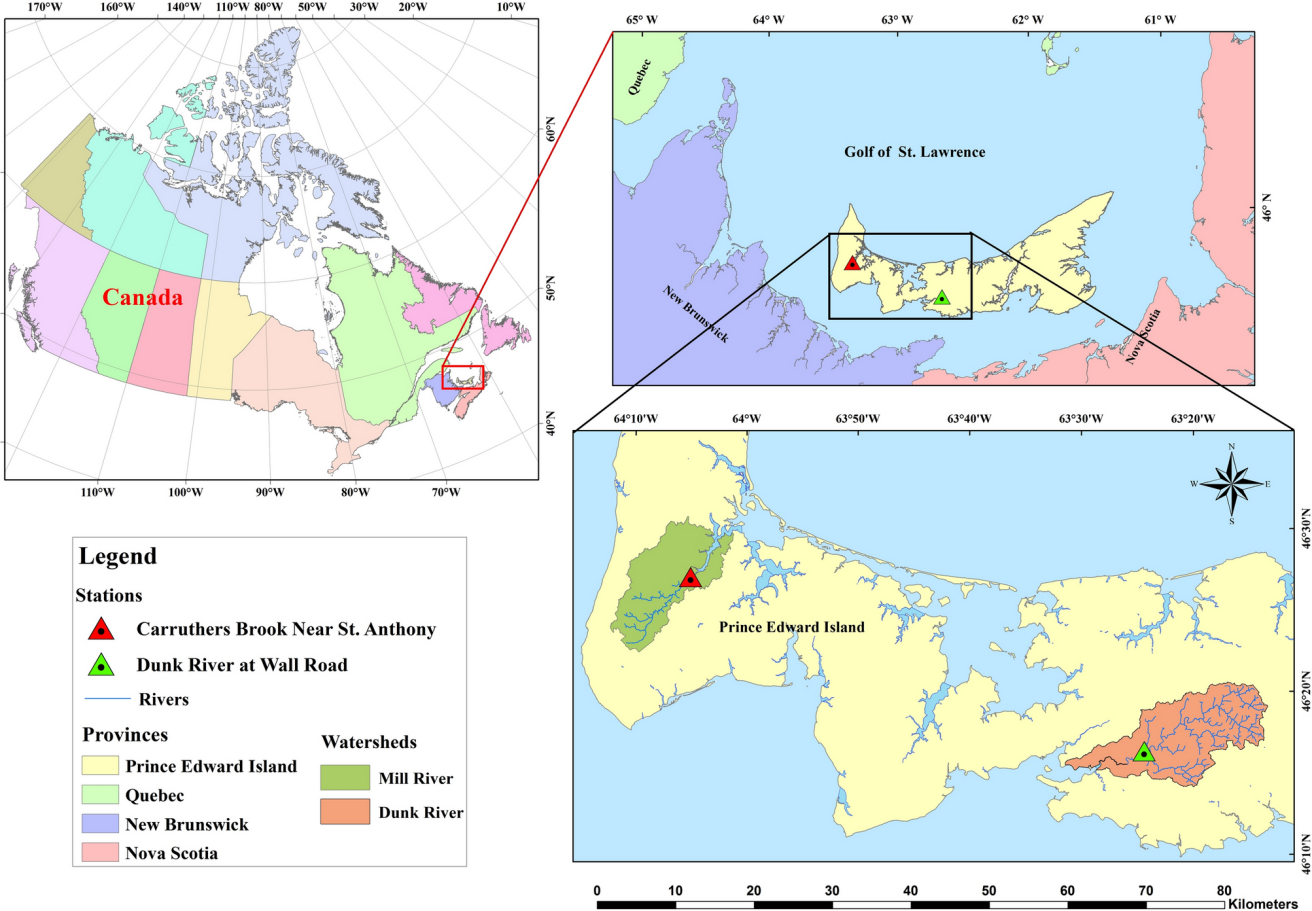
Geographic location of the Brook and Dunk Rivers stations in PEI province of Canada, developed by ArcMap^53^.

The main aim of the present research is to evaluate the efficiency and ability of MLMs to forecast water levels in multiple steps in the future for two rivers, Brook and Dunk, situated in the Prince Edward Island (PEI) province near the Gulf of Saint Lawrence in Canada. Figure 10 shows the geographical location of the Brook and Dunk Rivers in PEI. The Carruthers Brook Station near St. Anthony (Station No. 01CA003) is located on Brook River at 46° 44′ 38″ N latitude and 64° 11′ 13″ W longitude and has a gross drainage area of 46.8 km2. The Dunk River Station at Wall Road (Station No. 01CB002) is on the Dunk River at a latitude of 46° 20′ 45″ N and longitude of 63° 38′ 00″ W, a gross drainage area of 114 km^2^ (https://wateroffice.ec.gc.ca/).

The Brook and Dunk Rivers on PEI are significant for their ecological, agricultural, and recreational aspects. Both rivers provide essential habitats for wildlife, facilitate local agriculture via water supply and drainage, and enhance PEI’s natural ecology. The Brook and Dunk rivers are significant for flood control, water quality management, and provide recreational activities like fishing and canoeing, therefore improving the surrounding community’s quality of life. Studies on the Brook and Dunk Rivers have shown problems with agricultural runoff, sedimentation, and nutrient leaching. Attempts at water level monitoring by the PEI Department of Environment, Energy and Climate Action show that phosphorus and nitrogen levels often surge during spring runoff, hence possibly degrading water quality and causing algal blooms^54,55^. Additionally, seasonal variations in water levels are prevalent, with heightened runoff occurring during spring thaw and intense precipitation, potentially leading to localized floods and impacting agricultural output^56,57^ by eroding the topsoil. Effective monitoring of water levels using modern techniques may enhance watershed management plans to safeguard PEI’s freshwater resources^58^.

According to an analysis of historical hourly meteorological reports and model reconstructions from 1980 to 2016, the climate of PEI is characterized by moderate summers, cold winters with snow and wind, and partly cloudy throughout the year (https://weatherspark.com). July is the hottest month of the year in PEI (average maximum temperature of 24 °C), and January is the coldest month with an average minimum temperature of − 9 °C. Rainfall in PEI occurs continuously throughout the year. October is the wettest month (average rainfall of 60 mm), and February is the driest month (average rainfall of 15 mm). There is significant seasonal variation in monthly snowfall in PEI. February has the highest snowfall, with an average snowfall of 142 mm. (https://weatherspark.com).

To develop a multi-step-ahead water level forecast model for the Brook and Dunk rivers, hydrological and meteorological parameters obtained from the stations in the study area were utilized as input data (from 2015 to 2019 with daily time-step). The parameters are water level (m) (WL), flow (m^3^/s) (F), heat degree days (°C) (HD), total precipitation (mm) (P), mean temperature (°C) (T), relative humidity (%) (H), and dew point temperature (°C) (DP). Table 3 presents the statistical summaries of the parameters employed to develop the ML model.

**Table 3:** Descriptive Statistics related to the hydro-meteorological two cases study.

	Metric	F (m3/s)	T (°C)	HD (°C)	P (mm)	DP (°C)	H (%)	WL(m)
Brook	Min	0.11	− 19.70	0.00	0.00	− 24.88	38.25	0.29
	Average	1.11	6.02	12.39	3.27	2.21	77.09	0.50
	Max	20.20	25.20	37.70	74.00	22.18	100.00	2.24
	Median	0.52	6.00	12.00	0.00	2.53	77.79	0.44
	SD	1.82	10.18	9.60	7.23	10.27	11.62	0.21
	Kur	24.73	2.06	2.01	21.95	2.20	2.63	10.45
	Skew	4.10	− 0.12	0.28	3.78	− 0.24	− 0.34	2.37
Dunk	Min	0.55	− 19.70	0.00	0.00	− 24.88	38.25	0.50
	Average	2.62	6.02	12.39	3.27	2.21	77.09	0.68
	Max	42.60	25.20	37.70	74.00	22.18	100.00	1.84
	Median	1.91	6.00	12.00	0.00	2.53	77.79	0.65
	SD	2.79	10.18	9.60	7.23	10.27	11.62	0.13
	Kur	71.47	2.06	2.01	21.95	2.20	2.63	13.74
	Skew	6.57	− 0.12	0.28	3.78	− 0.24	− 0.34	2.34

For the Brook River, the data sets of T (°C), HD (°C), DP (°C), and H (%) collected from Carruthers Brook Station have distributions of approximately symmetric (-0.34 < Skewness < 0.28) and close to normal (2.01 < kurtosis < 2.63). Also, at the Brook River, the data sets of the Total Precipitation (mm), Flow (m^3^/s), and Level (m) are extremely skewed (Skewness values > 1) and with high positive values of Kurtosis (> 3) indicating Leptokurtic distribution (Table 3).

**Fig. 11:**
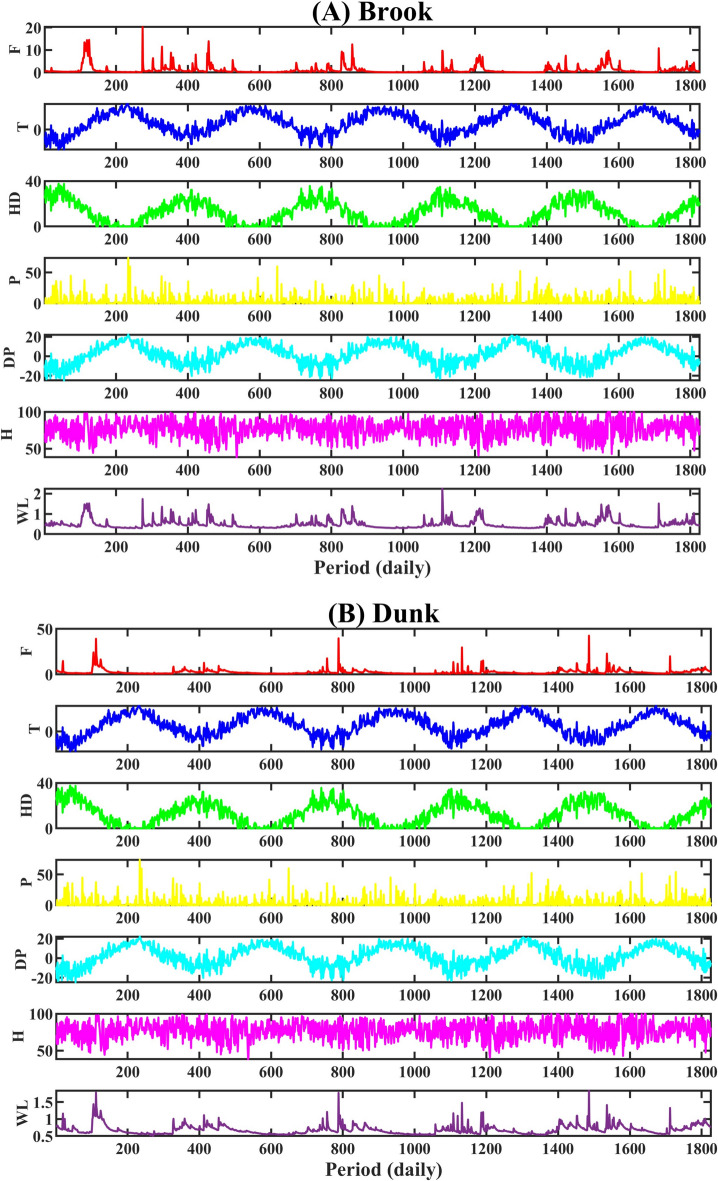
Time series graph of all date sets for (**A**) Brook and (**B**) Dunk.

For the Dunk River at the Dunk River Station, the data sets of T (°C), HD (°C), DP (°C), and H (%) with skewness values between [− 0.5, 0.5] are nearly symmetrical and close to normal distributions (0 < Kurtosis < 3). The data sets of Total Precipitation (mm), Flow (m^3^/s) and Level (m) with Skewness values [6.57, 2.34] are highly skewed and have Leptokurtic distributions (Kurtosis > 3) at the Dunk River Station (Table 3). Figure 11 demonstrates the original signals related to the input features and the water level in two cases study.

## Result and discussion

The standalone version of the models L-SKRidge, dRVFL, LASSO, KRidge, CFNN, and their MVMD-based hybrid version to forecast WL at (t + 1) and (t + 3) employing a set of metrics to measure and examine the performance accuracy in Brook and Dunk River Canada. The outcomes are presented mainly in tabular form as well as different ranges of diagnostic plots to evaluate these models for forecasting purposes.

Tables 4 and 5 demonstrate water level (WL) forecasting for Brook River to evaluate the performance of the standalone L-SKRidge, dRVFL, LASSO, KRidge, and CFNN models (Table 4) and their MVMD-based hybrid version (Table 5) at (t + 1) and (t + 3). The MVMD coupled with L-SKRidge model outperforms hybrid and standalone models and appears to be the most precise forecasting method based on (R = 0.971, RMSE = 0.054, NSE = 0.940, MaxAE = 0.484, U_95%_ = 0.148)-train and (R = 0.970, RMSE = 0.051, NSE = 0.937, MaxAE = 0.411, U_95%_ = 0.142)-test at (t + 1), followed by MVMD-based KRidge, CFNN, dRVFL, and LASSO models. The MVMD-based L-SKRidge model was better at predicting WL at (t + 3) than other MVMD-based and stand-alone methods used to predict WL at Brook River (Tables 4 and 5). Furthermore, the MVMD-based L-SKRidge model performed better for Brook River for both forecasting horizons (t + 1) and (t + 3) than other standalone and MVMD based methods (Table 4 and 5). It is also noteworthy that the hybrid models were better than their standalone counterpart models revealing better forecasting ability. The forecasting improvement in MVMD based L-SKRidge model based on NSE can be seen up to 12% at (t + 1) and 33% at (t + 3). Moreover, a significant increase in accuracy can be seen in other MVMD-based dRVFL, LASSO, KRidge, and CFNN methods compared with the standalone version.

**Table 4:** Performance of the standalone L-SKRidge, dRVFL, LASSO, KRidge, and CFNN models based on assessment metrics in Brook River.

	Methods	Mode	R	RMSE	MAPE	NSE	I_A_	MaxAE	U_95%_
WL (t + 1)	L-SKRidge	Train	0.903	0.094	7.880	0.816	0.946	1.624	0.262
		Test	0.893	0.092	8.145	0.797	0.941	0.915	0.254
	dRVFL	Train	0.853	0.115	11.799	0.728	0.915	1.603	0.318
		Test	0.853	0.110	11.030	0.706	0.897	1.070	0.303
	LASSO	Train	0.844	0.118	11.616	0.712	0.909	1.707	0.327
		Test	0.857	0.109	10.513	0.711	0.898	1.066	0.301
	KRidge	Train	0.888	0.101	10.016	0.787	0.937	1.562	0.281
		Test	0.873	0.099	9.875	0.762	0.926	1.051	0.275
	CFNN	Train	0.893	0.099	8.905	0.797	0.939	1.676	0.275
		Test	0.881	0.096	9.131	0.775	0.934	1.008	0.267
WL (t + 3)	L-SKRidge	Train	0.756	0.144	13.715	0.571	0.839	1.685	0.400
		Test	0.726	0.141	12.778	0.519	0.818	1.177	0.389
	dRVFL	Train	0.705	0.156	15.763	0.497	0.804	1.698	0.433
		Test	0.705	0.146	14.376	0.482	0.788	1.157	0.403
	LASSO	Train	0.702	0.157	15.716	0.492	0.800	1.682	0.435
		Test	0.705	0.146	14.148	0.483	0.788	1.159	0.403
	KRidge	Train	0.718	0.154	18.032	0.507	0.812	1.707	0.427
		Test	0.712	0.143	15.275	0.506	0.804	1.161	0.396
	CFNN	Train	0.726	0.152	16.633	0.524	0.828	1.558	0.420
		Test	0.686	0.149	15.249	0.463	0.799	1.155	0.412

**Table 5:** Performance of the hybrid MVMD based L-SKRidge, dRVFL, LASSO, KRidge, and CFNN models based on assessment metrics in Brooke River.

	Methods	Mode	R	RMSE	MAPE	NSE	I_A_	MaxAE	U_95%_
WL (t + 1)	L-SKRidge	Train	0.971	0.054	7.371	0.940	0.984	0.484	0.148
		Test	0.970	0.051	6.309	0.937	0.982	0.411	0.142
	dRVFL	Train	0.951	0.068	8.532	0.905	0.975	0.938	0.188
		Test	0.963	0.066	7.860	0.896	0.968	0.469	0.176
	LASSO	Train	0.948	0.070	8.553	0.899	0.973	0.961	0.194
		Test	0.960	0.066	8.022	0.895	0.968	0.465	0.178
	KRidge	Train	0.951	0.072	10.631	0.893	0.971	0.925	0.194
		Test	0.963	0.061	8.362	0.909	0.972	0.435	0.170
	CFNN	Train	0.964	0.059	8.123	0.928	0.981	0.856	0.163
		Test	0.955	0.062	8.527	0.907	0.973	0.380	0.172
WL (t + 3)	L-SKRidge	Train	0.967	0.056	7.019	0.935	0.983	0.355	0.156
		Test	0.928	0.079	8.664	0.849	0.957	0.535	0.215
	dRVFL	Train	0.916	0.089	11.357	0.838	0.954	1.109	0.245
		Test	0.912	0.088	10.735	0.813	0.942	0.692	0.240
	LASSO	Train	0.912	0.090	11.648	0.831	0.952	1.128	0.251
		Test	0.915	0.086	10.445	0.820	0.944	0.706	0.236
	KRidge	Train	0.914	0.093	13.404	0.823	0.950	1.094	0.253
		Test	0.916	0.084	10.943	0.831	0.948	0.675	0.232
	CFNN	Train	0.932	0.081	10.000	0.864	0.964	1.068	0.224
		Test	0.882	0.096	11.026	0.776	0.935	0.636	0.266

Tables 6 and 7 illustrate the efficacy of the MVMD-based hybrid and solo models in predicting WL for Dunk River at (t + 1) and (t + 3). Analysis of Tables 6 and 7 demonstrates that the MVMD-based L-SKRidge model consistently achieved the best precision in forecasting WL at both (t + 1) and (t + 3), as shown in the tables previously mentioned, in comparison to other models. The MVMD-based L-SKRidge model achieved superior performance metrics, with training scores of R = 0.975, RMSE = 0.030, MAPE = 2.690, NSE = 0.951, and MaxAE = 0.238, and testing scores of R = 0.958, RMSE = 0.039, MAPE = 3.371, NSE = 0.914, and MaxAE = 0.275, for forecasting WL at (t + 1).

**Table 6:** Performance of the standalone L-SKRidge, dRVFL, LASSO, KRidge, and CFNN models based on assessment metrics in Dunk River.

	Methods	Mode	R	RMSE	MAPE	NSE	I_A_	MaxAE	U_95%_
WL (t + 1)	L-SKRidge	Train	0.894	0.060	3.587	0.799	0.940	0.972	0.167
		Test	0.876	0.065	3.660	0.762	0.927	0.725	0.179
	dRVFL	Train	0.821	0.077	5.864	0.674	0.893	1.048	0.212
		Test	0.755	0.090	5.845	0.549	0.846	0.968	0.246
	LASSO	Train	0.804	0.080	6.072	0.647	0.881	1.071	0.221
		Test	0.757	0.090	5.975	0.544	0.838	0.920	0.246
	KRidge	Train	0.871	0.068	5.263	0.746	0.921	1.026	0.185
		Test	0.850	0.071	4.506	0.719	0.907	0.789	0.196
	CFNN	Train	0.888	0.062	4.057	0.786	0.937	0.958	0.171
		Test	0.867	0.068	4.395	0.743	0.923	0.662	0.186
WL (t + 3)	L-SKRidge	Train	0.789	0.082	5.636	0.622	0.866	1.084	0.228
		Test	0.727	0.094	5.387	0.506	0.816	1.093	0.257
	dRVFL	Train	0.721	0.093	7.319	0.520	0.816	1.067	0.257
		Test	0.655	0.105	7.489	0.387	0.763	0.963	0.285
	LASSO	Train	0.709	0.095	7.259	0.502	0.804	1.096	0.262
		Test	0.653	0.105	7.220	0.382	0.754	1.021	0.286
	KRidge	Train	0.779	0.084	5.850	0.606	0.857	1.074	0.233
		Test	0.713	0.096	5.852	0.481	0.799	1.080	0.263
	CFNN	Train	0.785	0.083	6.098	0.615	0.872	1.027	0.230
		Test	0.678	0.100	6.612	0.434	0.798	1.094	0.277

**Table 7:** Performance of the hybrid MVMD based L-SKRidge, dRVFL, LASSO, KRidge, and CFNN models based on assessment metrics in Dunk River.

	Methods	Mode	R	RMSE	MAPE	NSE	I_A_	MaxAE	U_95%_
WL (t + 1)	L-SKRidge	Train	0.975	0.030	2.690	0.951	0.987	0.238	0.082
		Test	0.958	0.039	3.371	0.914	0.976	0.275	0.108
	dRVFL	Train	0.936	0.047	4.374	0.876	0.966	0.345	0.131
		Test	0.905	0.060	5.168	0.801	0.941	0.392	0.162
	LASSO	Train	0.933	0.048	4.386	0.870	0.964	0.361	0.134
		Test	0.908	0.059	5.080	0.806	0.943	0.398	0.160
	KRidge	Train	0.937	0.049	4.872	0.867	0.964	0.362	0.133
		Test	0.914	0.054	4.725	0.834	0.951	0.387	0.151
	CFNN	Train	0.929	0.051	4.752	0.854	0.961	0.530	0.140
		Test	0.911	0.060	5.268	0.800	0.948	0.510	0.161
WL (t + 3)	L-SKRidge	Train	0.951	0.041	3.628	0.905	0.974	0.322	0.115
		Test	0.915	0.054	4.637	0.837	0.954	0.397	0.150
	dRVFL	Train	0.907	0.057	5.104	0.822	0.949	0.498	0.157
		Test	0.880	0.066	5.466	0.757	0.924	0.499	0.180
	LASSO	Train	0.906	0.057	5.145	0.821	0.949	0.493	0.157
		Test	0.881	0.065	5.441	0.761	0.926	0.501	0.178
	KRidge	Train	0.934	0.048	4.117	0.869	0.962	0.491	0.134
		Test	0.885	0.064	4.857	0.769	0.928	0.536	0.176
	CFNN	Train	0.910	0.056	5.333	0.824	0.952	0.422	0.155
		Test	0.877	0.066	5.650	0.759	0.932	0.456	0.180

The other MVMD-based hybrid versions of the models were also reasonably good in accuracy but could not surpass the MVMD-based L-SKRidge model, while the performance of standalone models was very lower. Similarly, the MVMD-based L-SKRidge model had the best level of accuracy in predicting WL for Dunk River at (t + 3) compared to other models (Tables 6 and 7). The MVMD-based hybrid models demonstrate superior performance relative to the solo models in predicting the WL at both forecast horizons. Overall, the MVMD-based L-SKRidge model demonstrated superior accuracy in forecasting WL compared to other models. A significant increase in accuracy has been seen by integrating the MVMD with the L-SKRidge model.

The present study uses the MARCOS approach as an MCDM method to choose the best appropriate model using a set of criteria. The MARCOS used seven statistical metrics (R, RMSE, MAPE, NSE, IA, MaxAE, and U_95%_) to select the best model. The MARCOS technique guarantees a comprehensive and equitable evaluation of model performance. It facilitates the discovery of the model that most effectively satisfies the specified requirements for accuracy and dependability.

**Fig. 12:**
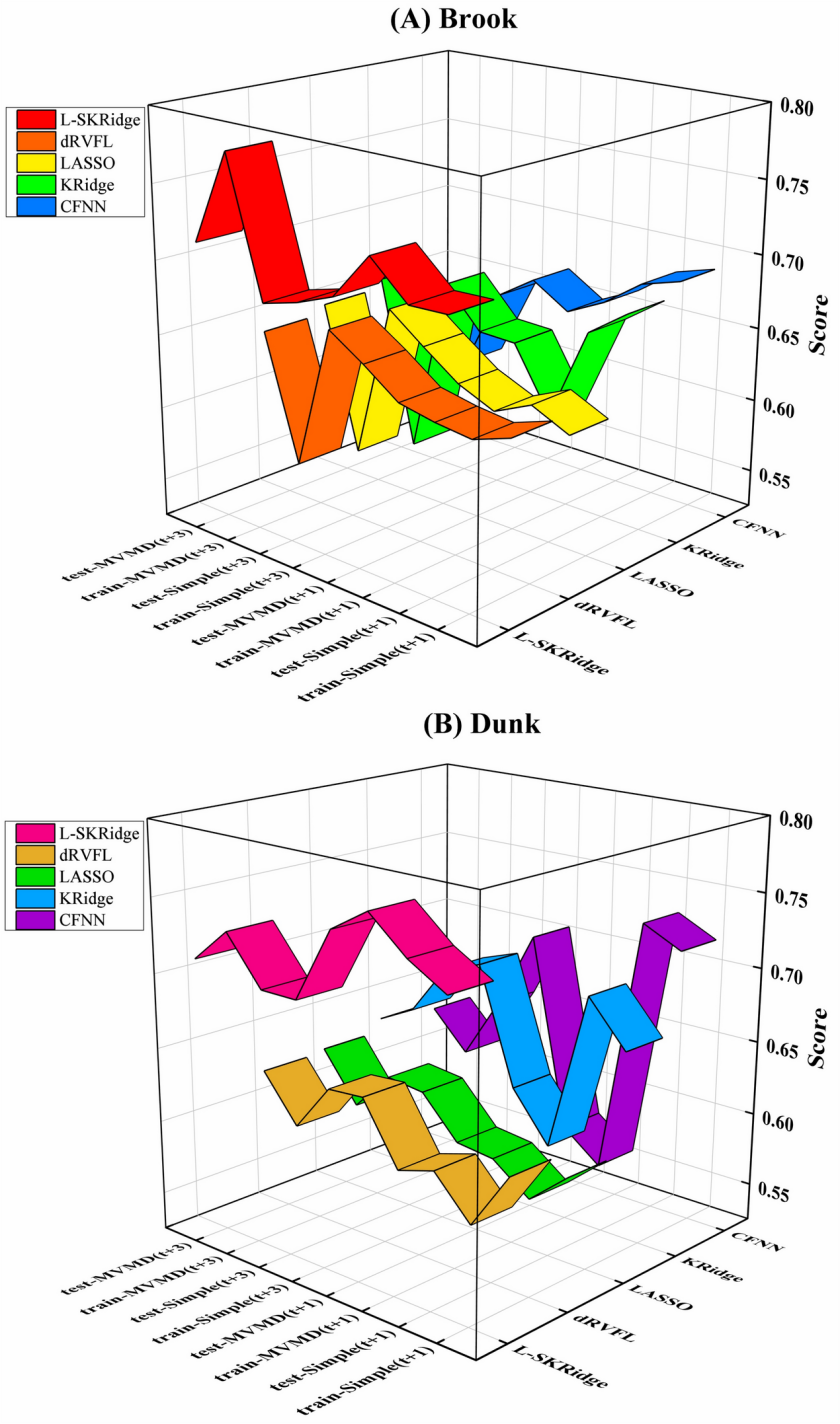
MARCOS scores achieved by five ML models over two-time horizons for simple and hybrid modes and in (**A**) Brooke and (**B**) Dunk River.

Figure 12 indicates the MARCOS Score attained by the hybrid MVMD-based and standalone L-SKRidge, dRVFL, LASSO, KRidge, and CFNN models during training and testing periods to predict WL at two time horizons ((t + 1) and (t + 3)) for Brooke and Dunk Rivers. The MVMD-based L-SKRidge method achieved an outstanding MARCOS score for Brooke River at both (t + 1) and (t + 3) in comparison to other ML methods.

Similarly, the top values of TOPSIS score were seen in Dunk River, which is evidence that the MVMD-based L-SKRidge model demonstrates a superior level of precision especially to forecasting WL at (t + 1) and (t + 3) compared with the other models. As a result, the MARCOS score suggests that the MVMD-based L-SKRidge model is superior to accurately estimate WL for Brooke and Dunk River.

**Fig. 13:**
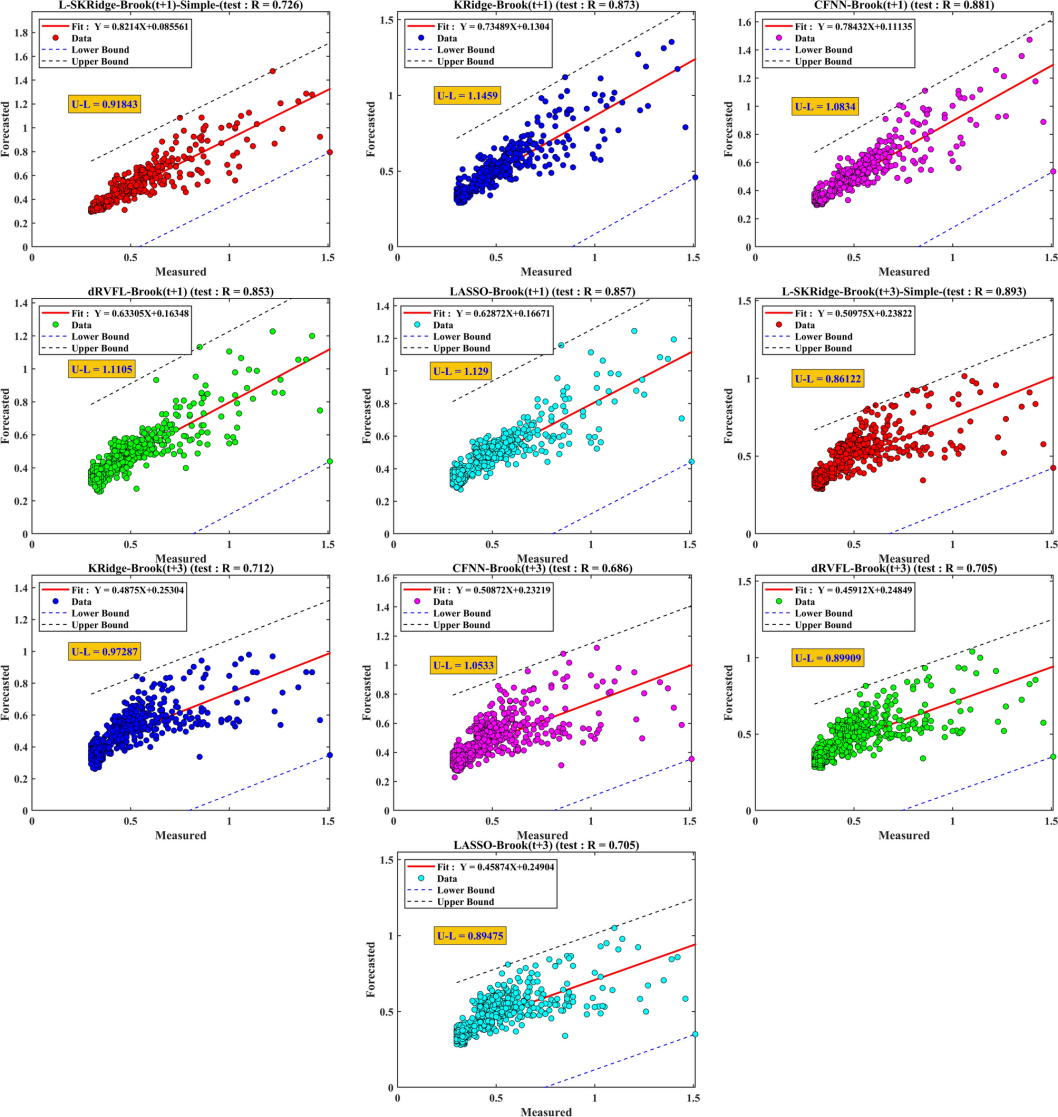
Scatter plots of all standalone ML models over two time horizons for Brook river.

Figure 13 is based on a scatter diagram to inspect the comparison between the measured and forecasted WL generated by the standalone ML-based L-SKRidge, dRVFL, LASSO, KRidge, and CFNN models in the testing period for Brook and Dunk River at (t + 1) and (t + 3). Moreover, the values of R metrics and fitted lines with equations is also incorporated along with lower and upper bounds. This research established upper and lower boundaries around the regression line to illustrate possible diversity in predictions. A best-fit line was produced by linear regression, illustrating the primary trend in the correlation between the measured and predicted values. The methodology determined the boundaries by examining the distance of individual data points from the line, which represents the unexplained variability not accounted for by the trend. The minimum and maximum deviations from the line were used to determine the limits, forming a lower and upper boundary around the best-fit line over the whole data range. These boundaries effectively provide a “buffer zone” around the projected values, signifying the range in which the actual data points are expected to reside. The interval width, shown as dashed lines on the graph, graphically represents the possible mistake in the predictions. Incorporating these limitations enhances the model’s prediction reliability and aids users in comprehending the variability margin in the projected values.

**Fig. 14:**
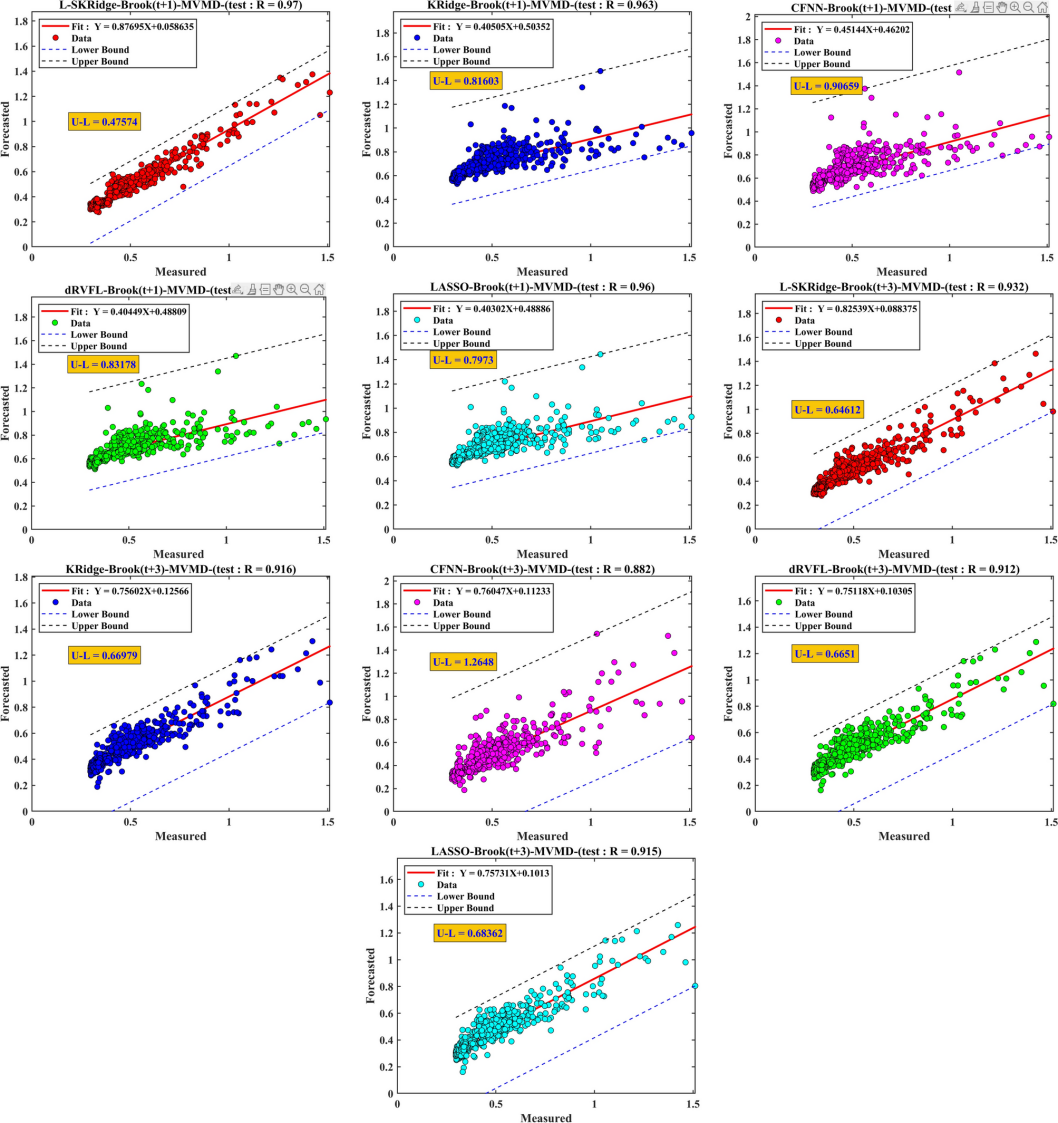
Scatter plots of all hybrid ML models over two time horizons for Brook river.

The standalone version of the models was relatively poor based on scatter plots in Fig. 13 compared to the MVMD-based methods (Fig. 14). From Fig. 13, the proposed L-SKRidge yields the best results with the smallest values of U–L (i.e., the difference between their lower and upper limits (U–L)) for two-time horizons (U–L(t + 1) ) = 0.91 and U–L(t + 3) = 0.86) compared with the other standalone models. Figure 14 presents a scatter map that examines the contrast between the observed and predicted water levels (WL) provided by several models, including the hybrid MVMD-based L-SKRidge, dRVFL, LASSO, KRidge, and CFNN models. This analysis is conducted throughout the testing period for the Brook and Dunk River at time points (t + 1) and (t + 3). The comparative analysis of prediction outcomes for two-time horizons reveals that the L-SKRidge model has superior performance compared to the dRVFL, LASSO, KRidge, and CFNN approaches in relation to the U–L. In the scenario involving WL for t + 1, it is seen that L-SKRidge demonstrates the narrowest discrepancy between the upper and lower bounds (U–L), with a value of 0.47. This value is comparatively less than those obtained for dRVFL (0.83), LASSO (0.79), KRidge (0.81), and CFNN (0.9), indicating L-SKRidge’s superior performance in minimizing the U–L gap. The model that has been suggested also exhibits the smallest difference between U and L for WL at t + 3, as seen in Fig. 13, with a numerical value of 0.64. The hybrid MVMD based L-SKRidge model at (t + 1) and (t + 3) outperformed to forecast WL in terms of R = 0.97, and 0.932 followed by MVMD based KRidge, LASSO, dRVFL, and CFNN models for Brook River. For Dunk River, the MVMD-based L-SKRidge model once again obtained better precision against other MVMD-based hybrid and standalone counterparts to forecast WL at both forecasting horizons (refer to Appendix C). Consequently, the scatter plots demonstrated that MVMD-based methods outperform solo variants in forecasting WL for both stations. The L-SKRidge model, based on MVMD, outperforms all other models in terms of accuracy. This performance is highly promising.

**Fig. 15:**
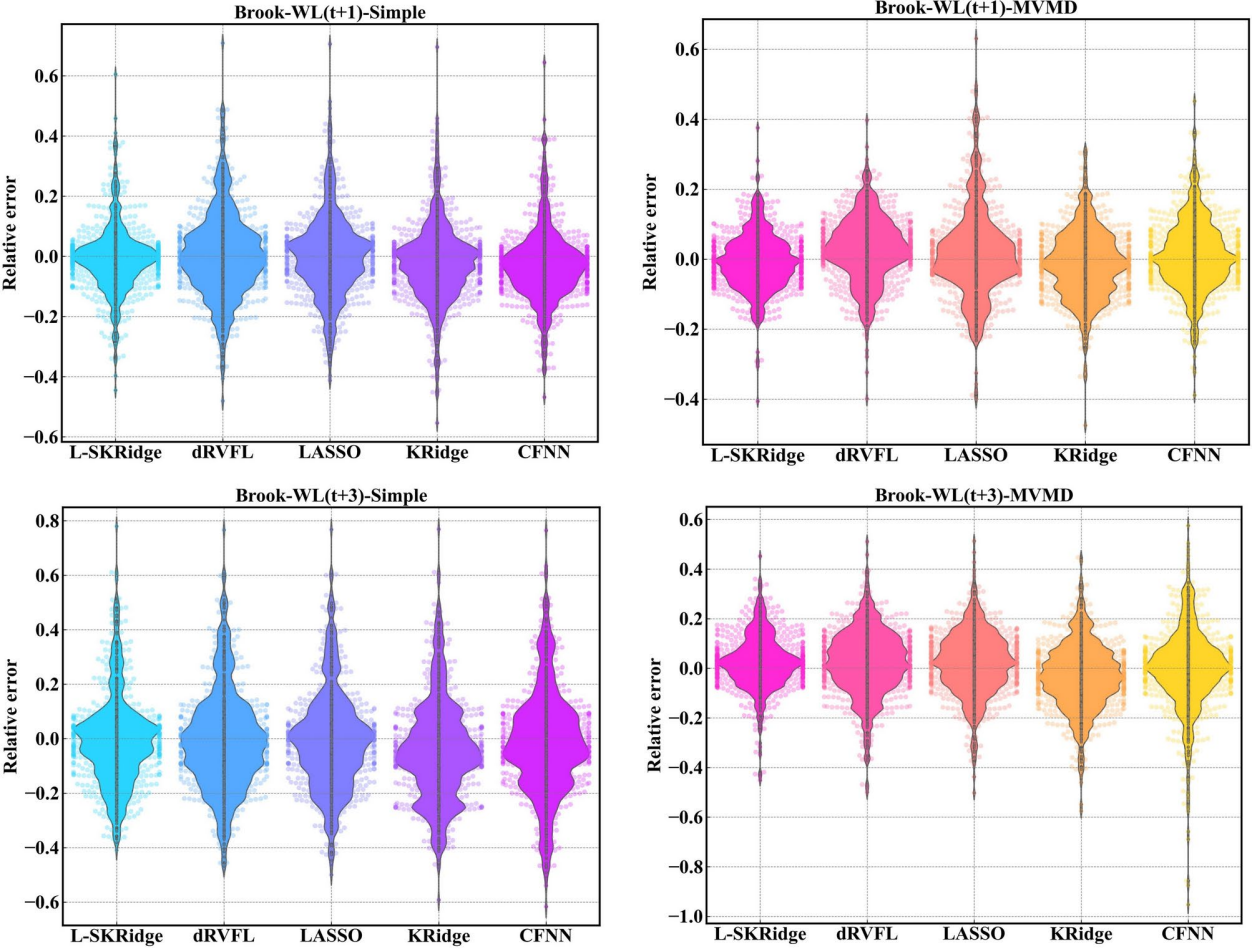
Violin plot of relative error for all ML models at two-time horizons in Brook River.

**Fig. 16:**
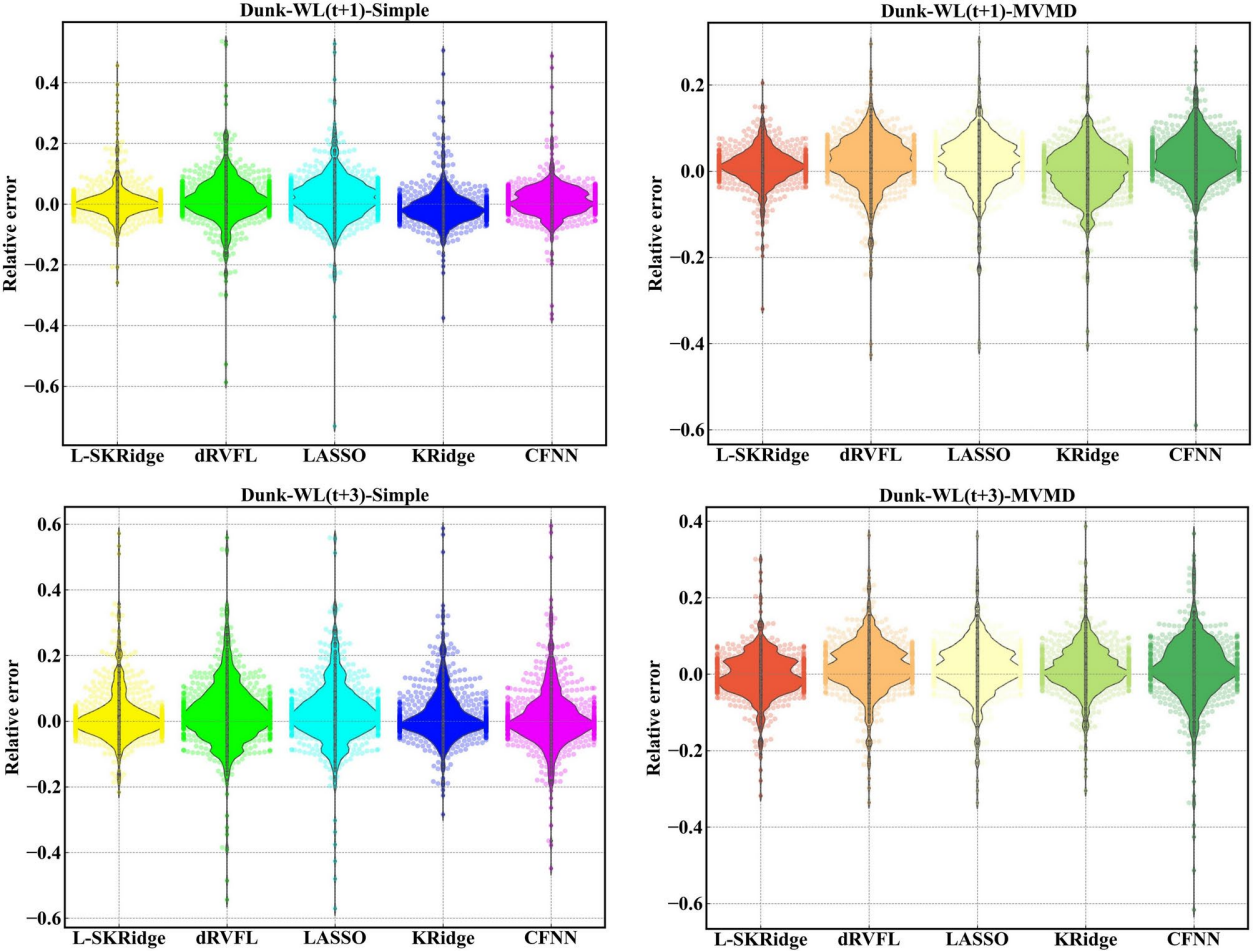
Violin plot of relative error for all ML models at two-time horizons in Brook River.

The violin plots in Fig. 15 provide a diagnostic assessment calculating the relative error of the forecasted WL for the MVMD-based models as well as standalone L-SKRidge, dRVFL, LASSO, KRidge, and CFNN models at (t + 1) and (t + 3). From the figure, the hybrid MVMD-based L-SKRidge clearly showed a smaller relative error and more accurate and consistent violin distribution (i.e., ranging between − 0.4 and + 0.4) at (t + 1) and (− 0.5 and + 0.5 at (t + 3) to forecast WL in Brook River compared with all other MVMD-based models. Similarly, the hybrid MVMD based L-SKRidge method appeared to be accurate for Dunk River using violin plot distributions based on relative error against other comparing models at both forecast horizons (see Fig. 16). Thus, MVMD based L-SKRidge model achieves better WL forecasting accuracy for both rivers at (t + 1) and (t + 3).

**Fig. 17:**
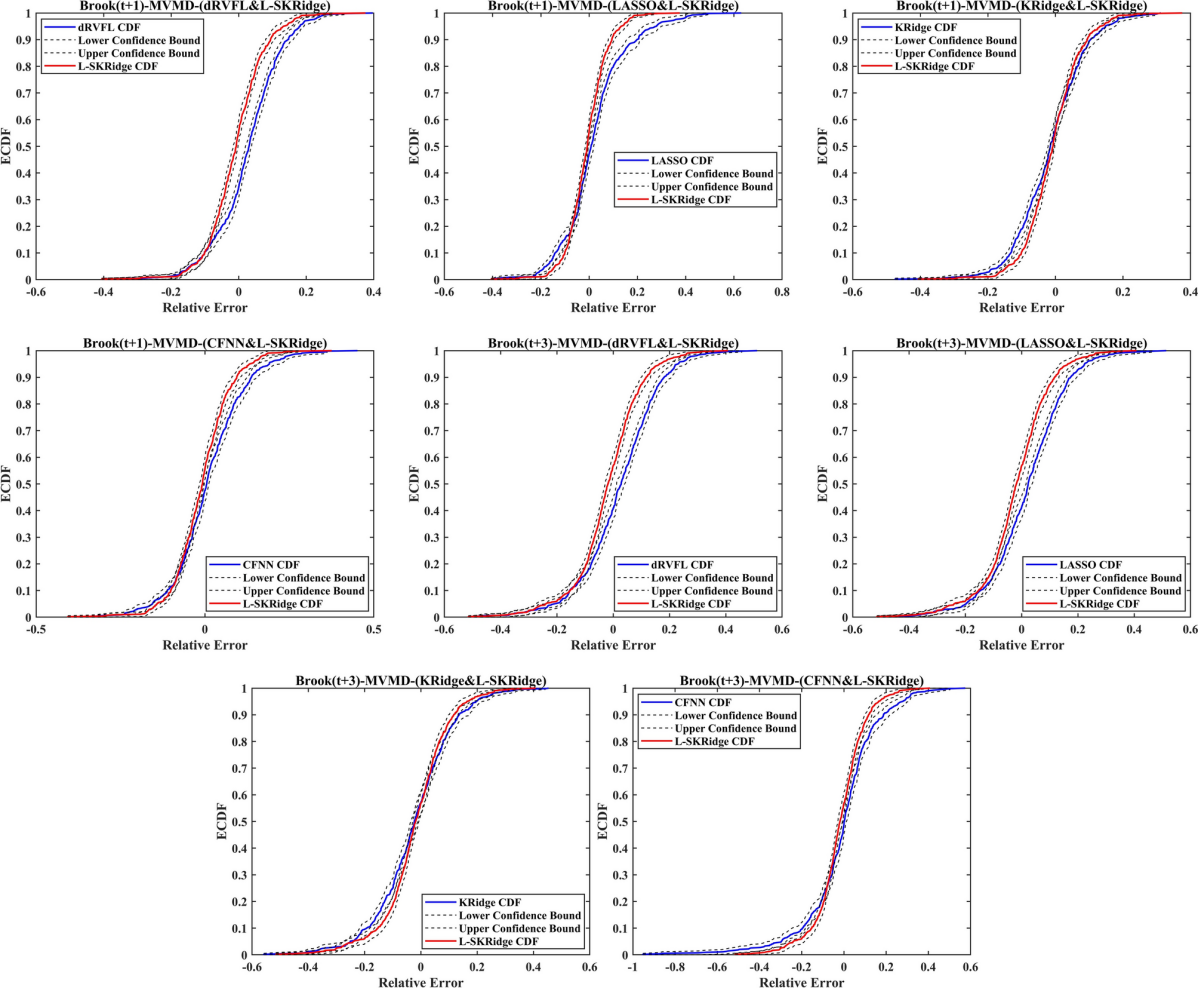
ECDF calculated using five ML methods at two-time horizons in Brook River.

**Fig. 18:**
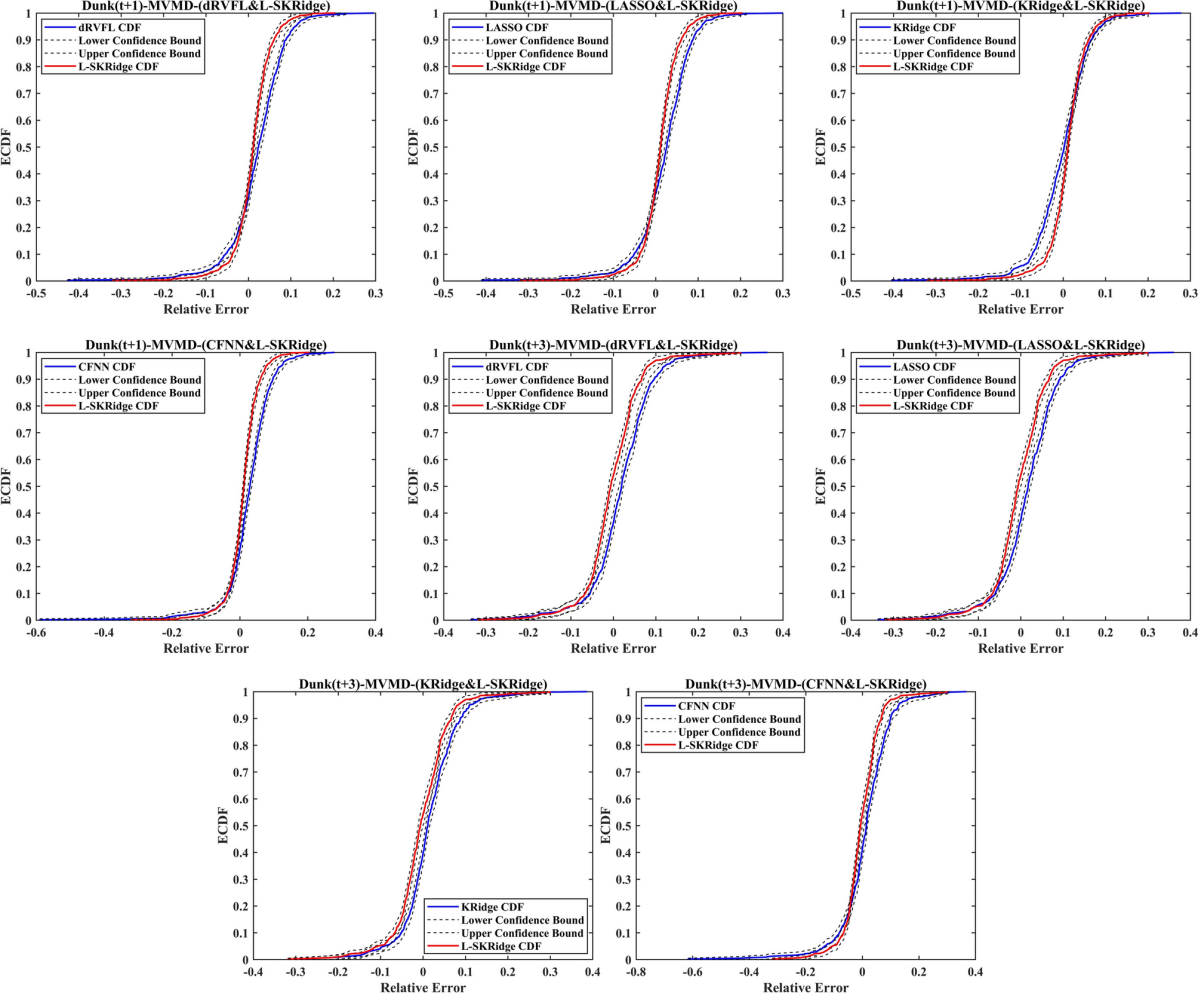
ECDF calculated using five ML methods at two-time horizons in Dunk River.

The forecasted WL of Brooke and Dunk Rivers are given in Figs. 17 and 18 using the empirical cumulative distribution function (ECDF) within the lower and upper confidence intervals to evaluate the precision of the MVMD-based L-SKRidge model to other models for a clearer representation of the results. The ECDF plot provides a cumulative view of relative errors, showing the proportion of data points below each error value. It helps assess overall model performance, reliability, and robustness by illustrating error accumulation. This visualization makes it easier to compare models and communicate performance effectively. By using the ECDF plot, you emphasize cumulative accuracy and provide a comprehensive analysis.

The ECDF of the MVMD-based L-SKRidge model for both Brooke and Dunk Rivers had a remarkably similar pattern at both (t + 1) and (t + 3) forecasting horizons compared to other MVMD-based dRVFL, LASSO, KRidge, and CFNN models. The analysis also depicts that the forecasts based on MVMD based L-SKRidge model are within the lower and upper confidence bounds to confirm the validity against other comparing models. Hence, these figures further authenticate the suitability of the MVMD-based L-SKRidge method to predict WL at the time horizons.

**Fig. 19:**
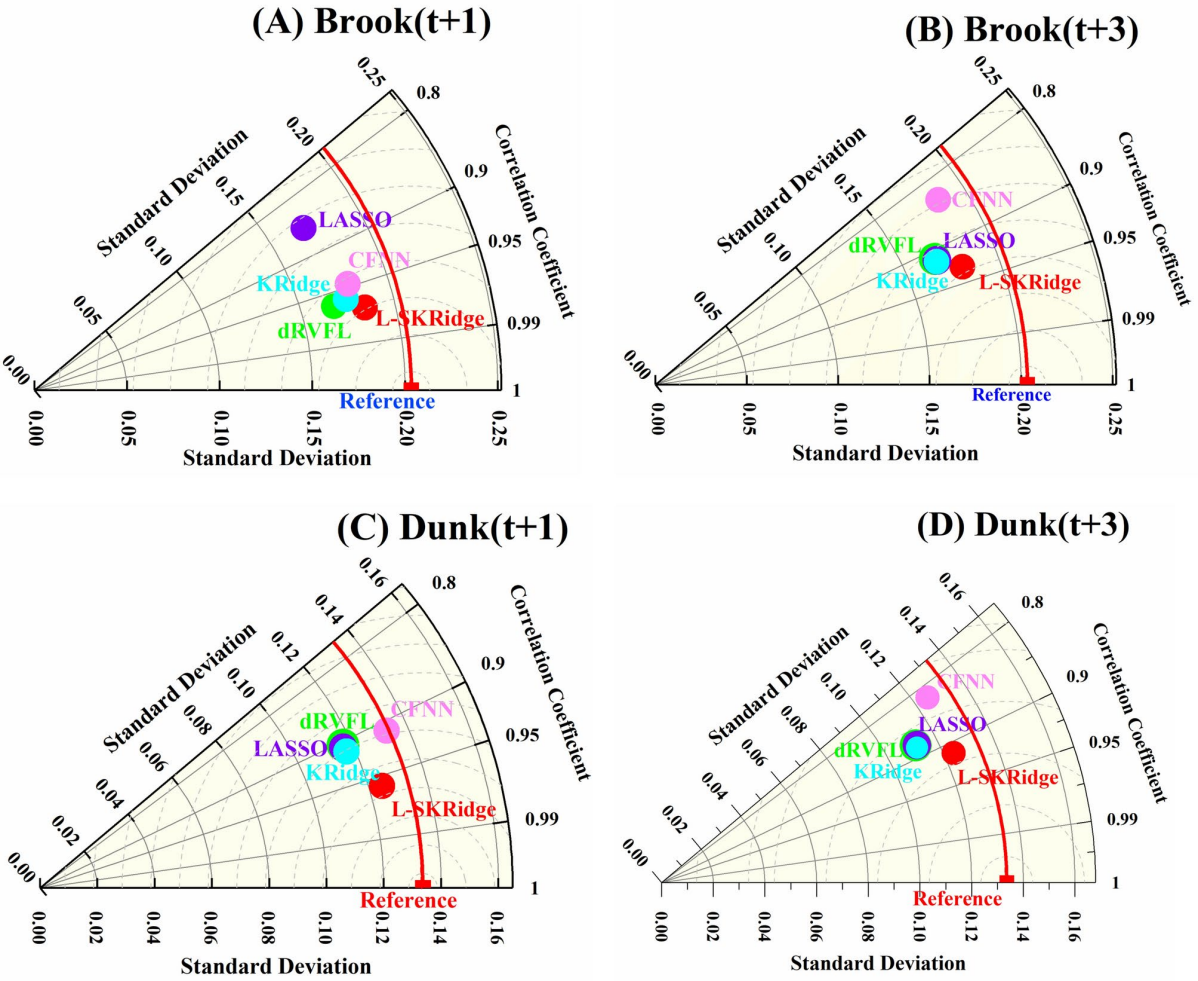
Taylor diagram of five ML models for two-time horizons in Brook and Dunk River.

The Taylor plots in Fig. 19 referred to the referenced and forecasted WL using MVMD-based L-SKRidge (red), dRVFL (green), LASSO (purple), KRidge (Cyan), and CFNN (pink) models at (t + 1) and (t + 3) to evaluate the precision for both Rivers. A comprehensive evaluation of the models’ comparability with respect to standard deviation and correlation coefficient is facilitated by these graphic design. For Brooke River, clearly the MVMD-based L-SKRidge positioned very closely to the reference WL with a correlation coefficient between 0.95 and 0.99 (t + 1) and 0.90–0.95 (t + 3) with a standard deviation (0.20– 0.25). The hybrid MVMD-based dRVFL, LASSO, KRidge, and CFNN comparing models are reasonably satisfactory but could not go ahead than the MVMD-based WKRidge model. Additionally, the MVMD-based L-SKRidge model achieved a superior rank in terms of accuracy for Dunk River in comparison to other models that were used to estimate WL at (t + 1) and (t + 3). The Taylor diagram thus supports the suitability for better WL forecasting of the MVMD-based L-SKRidge model.

## Conclusion

A new hybrid model called SKRidge has been created by combining the effective characteristics of SVD and KRidge. Additionally, the model incorporates MVMD and employs LGBM as an astute feature selection method, supplemented by the application of the RUN algorithm for parameter tuning. Furthermore, the SKRidge, combined with ridge regression and incorporating a linear relationship, has made a significant contribution to the formulation of the proposed model, known as L-SKRidge, with the specific objective of enhancing forecasting accuracy. The main objective of this research is to forecast water levels in Canada’s Brook and Dunk Rivers for two timeframes.

The proposed framework exhibits the following critical features:

- *Hybridization of SKRidge and Ridge regression*: L-SKRidge uses the capabilities of SKRidge and ridge regression. This combination considerably increases the overall model performance. It is especially focused on improving predicting accuracy.
- *MVMD method*: The model leverages MVMD for the decomposition of input variables into IMFs, and utilizes PACF to identify influential lags at both (t + 1) and (t + 3).
- *Feature selection with importance value factor*: The proposed framework employs an efficient feature selection (LGBM) to identify the most influential input datasets. The selected features are incorporated into the L-SKRidge model to improve its predictive abilities.
- *Comparative analysis with other models*: To assess the forecasting accuracy and identify MVMD-L-SKRidge’s ability, the MVMD is combined with other models (dRVFL, LASSO, KRidge, and CFNN).

Summary of research results:

The study findings provide a comprehensive evaluation of several forecasting models for predicting water levels in the Canadian rivers Dunk and Brook. Here are the key findings and numerical outcomes:

i. Brook river forecasting results at (t + 1):
  L-SKRidge exhibited superior precision with an R value of 0.97, RMSE of 0.054, MAPE of 7.371, NSE of 0.940, IA of 0.984, MaxAE of 0.484, and U95% of 0.148 in training.
  For testing, L-SKRidge achieved an R value of 0.970, RMSE of 0.051, MAPE of 6.309, NSE of 0.937, IA of 0.982, MaxAE of 0.411, and U95% of 0.142.
ii. Brook river forecasting results at (t + 3):
  L-SKRidge continued to outperform other models with high accuracy, surpassing both standalone and hybrid versions.
iii. Dunk river forecasting results:
  L-SKRidge maintained its superiority, exhibiting higher accuracy for forecasting at (t + 1) and (t + 3) compared to other models, including the standalone versions.
iv. Forecasting improvement:
  The integration of MVMD with WKRidge resulted in a notable increase in forecasting accuracy, with NSE improvements of up to 12% at (t + 1) and 33% at (t + 3).
v. Comparative performance:
  The hybrid models consistently outperformed their standalone counterparts, underlining their improved forecasting abilities.
vi. Taylor plots and ECDF analysis:
  Taylor plots revealed the high precision of L-SKRidge, with correlation coefficients ranging from 0.90 to 0.99 and standard deviations of 0.20 to 0.25 for Brook River.
  ECDF analysis confirmed the consistency of MVMD-based L-SKRidge forecasts within the lower and upper confidence bounds.

In summary, the research showcases the exceptional forecasting capacity of the proposed MVMD-based L-SKRidge model, which consistently outperforms other models across various evaluation metrics, and offers a substantial advancement in water level prediction accuracy for the Brook and Dunk Rivers.

The proposed MVMD-based L-SKRidge model exhibits enhanced forecasting precision, particularly for water level predictions in the Brook and Dunk Rivers. The main advantages of this method are the development of an efficient model, the decomposition of inputs into effective signals provided by MVMD, and the selection of specific features through LGBM, all of which increase the prediction accuracy. The suggested approach exhibits great forecasting accuracy for water level predictions; nonetheless, practical obstacles persist. The main concerns are the computational complexity of the model, which requires significant resources, and the over-sensitivity of the fit resulting from fine-tuning of the parameters. Scalability is an important issue, as the model may require a more powerful optimization for use on larger datasets or diverse contexts.

Further studies should focus on broadening the model’s application to forecast in other domains, such as climate change impacts, renewable energy trends, and agricultural forecasts, which might significantly enhance its relevance and utility. Validation in these new areas may improve the model and uncover any specific requirements or modifications needed. Moreover, integrating the L-SKRidge model into a comprehensive decision-support framework might enhance its practical use. Real-time forecasts and integration with geographic information systems (GIS) might improve decision-making tools for policymakers and stakeholders in water resource management and other domains.

## Supplementary Information

Supplementary Information.

## Data Availability

Data availability The data that support the findings of this study are available from the corresponding author upon reasonable request.
