# Supplementary material for: A hybrid framework: singular value decomposition and kernel ridge regression optimized using mathematical-based fine-tuning for enhancing river water level forecasting
Source: Sci Rep. 2025 Mar 4;15:7596. doi: 10.1038/s41598-025-90628-6 (PMC11880535; doi:10.1038/s41598-025-90628-6)
Supplement: Supplementary file 1 — Supplementary Information. [file 41598_2025_90628_MOESM1_ESM.docx]

**Appendix A: Results of LGBM-FS for Dunk River**

|  |  |
| --- | --- |
| 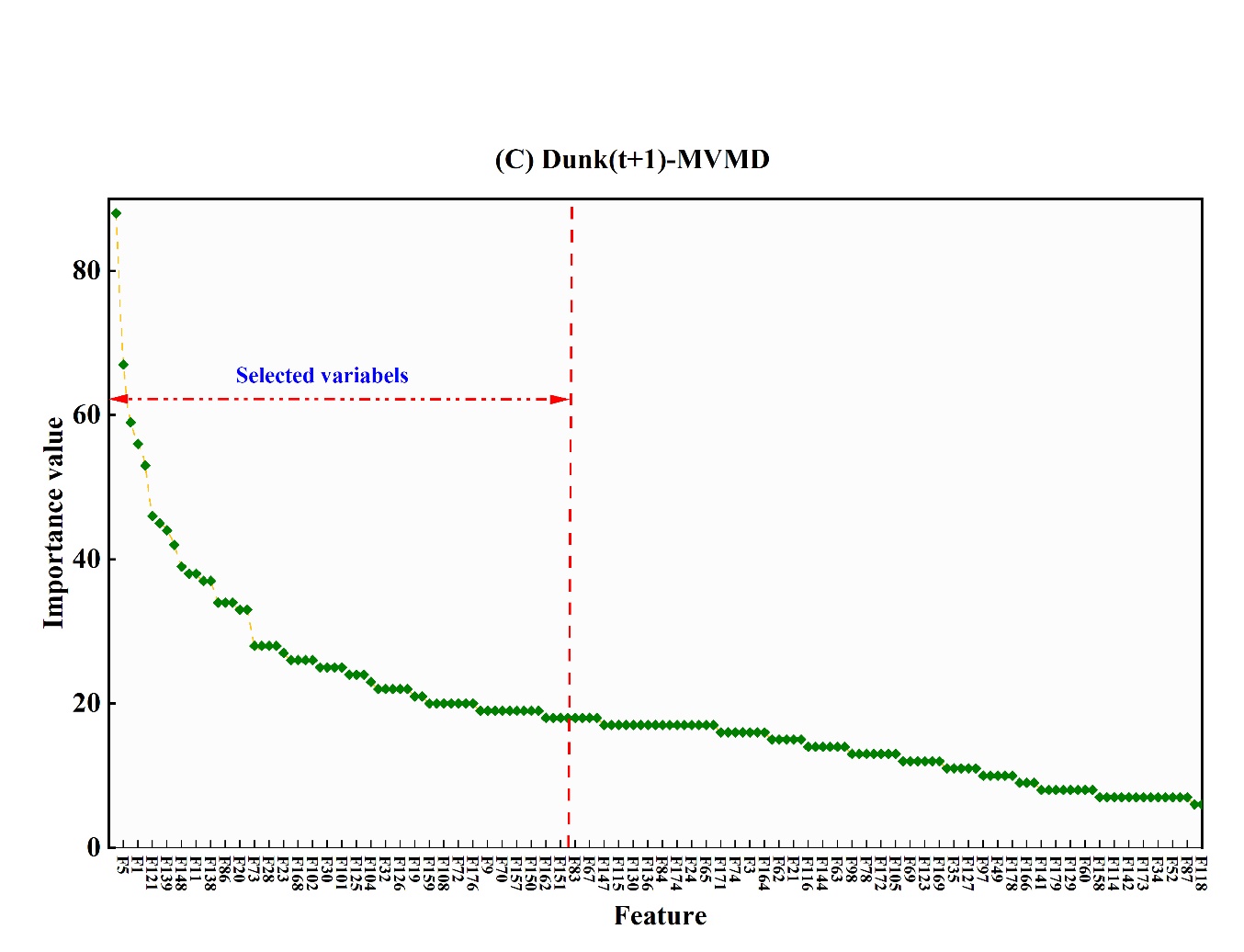 | |
| 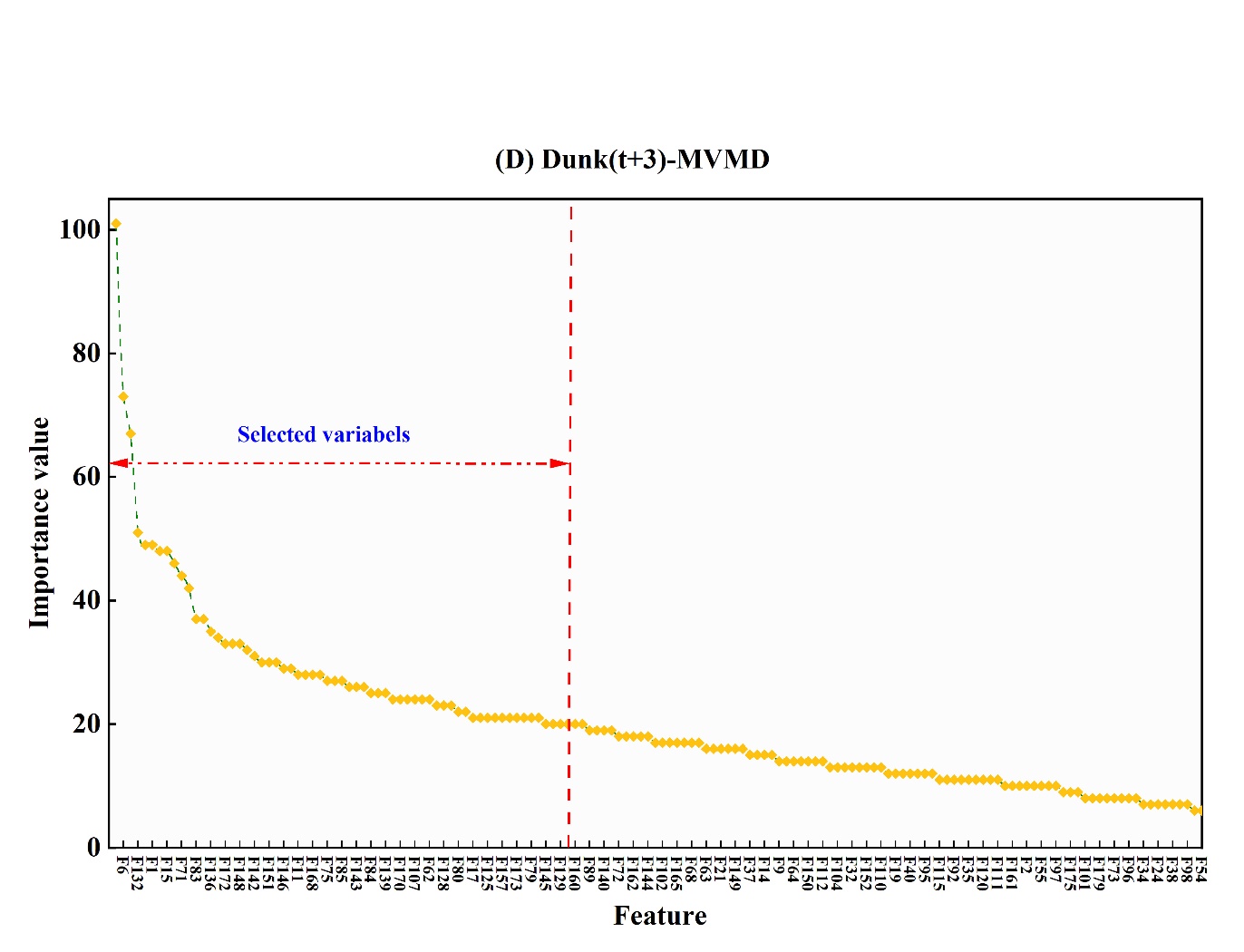 | |
| **Fig. A.** Outcomes of the LGBM-FS scheme on the most effective sub-components extraction among available pool of decomposed sub-sequences in every horizon for Dunk river | |

**Appendix B: ACF plot**

| 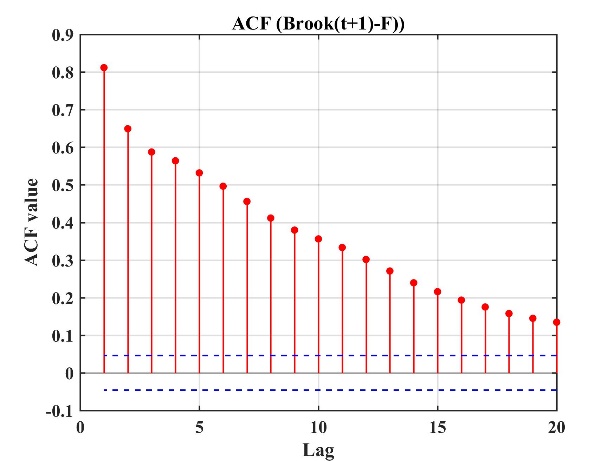 | 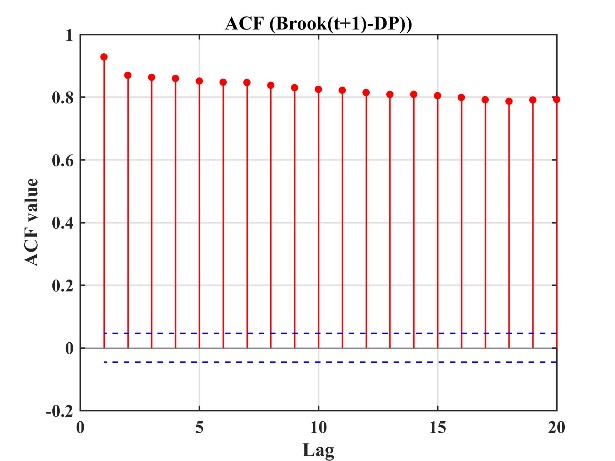 | 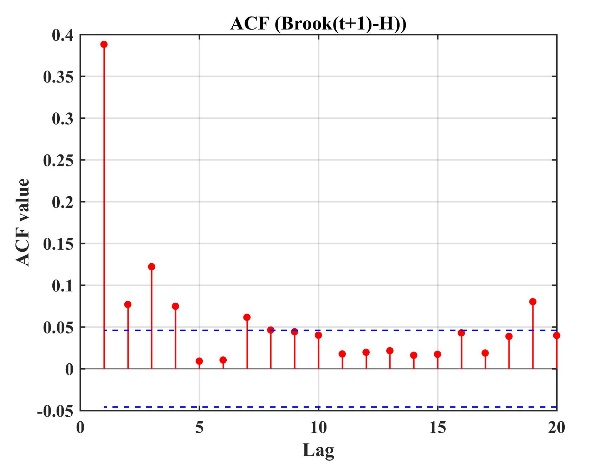 |
| --- | --- | --- |
| 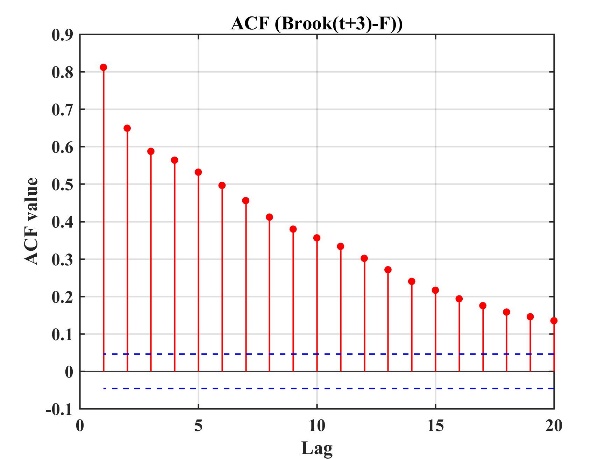 | 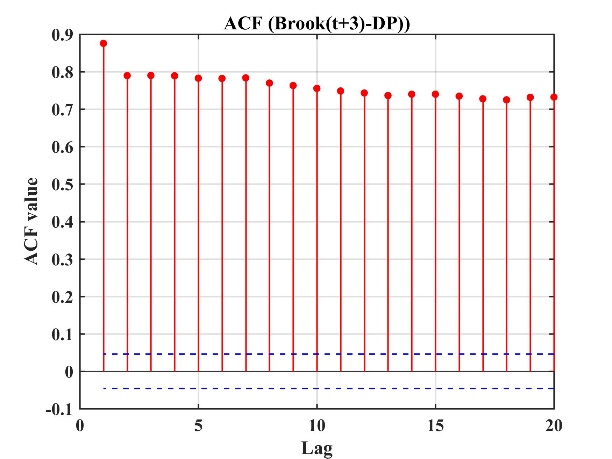 | 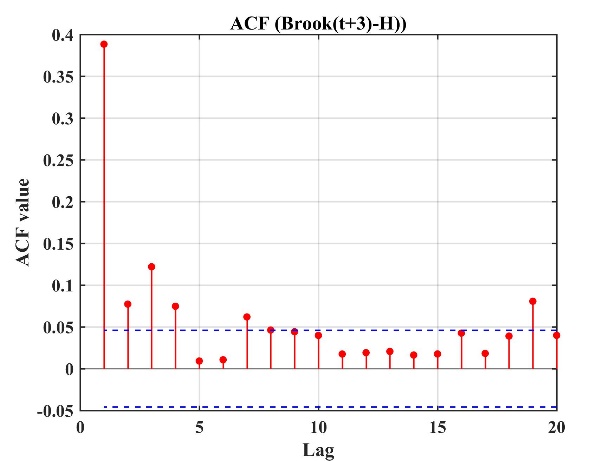 |
| 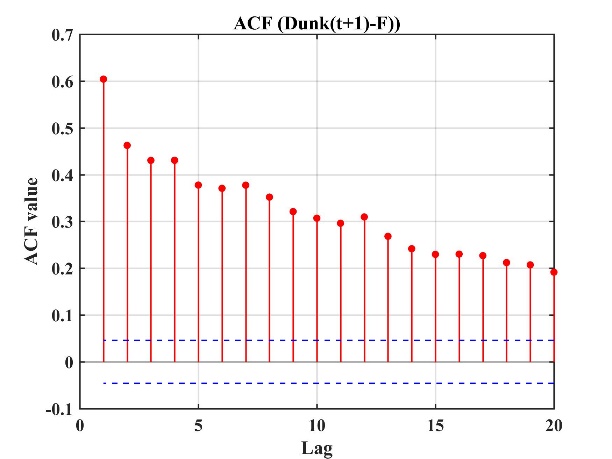 | 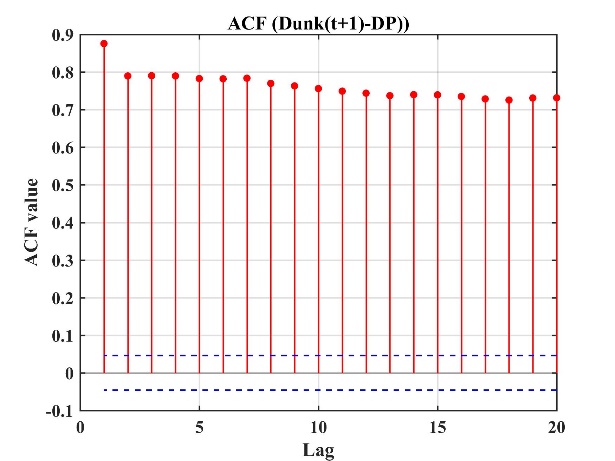 | 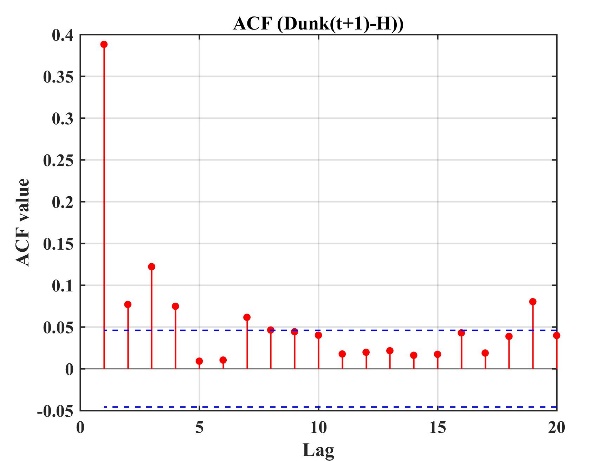 |
| 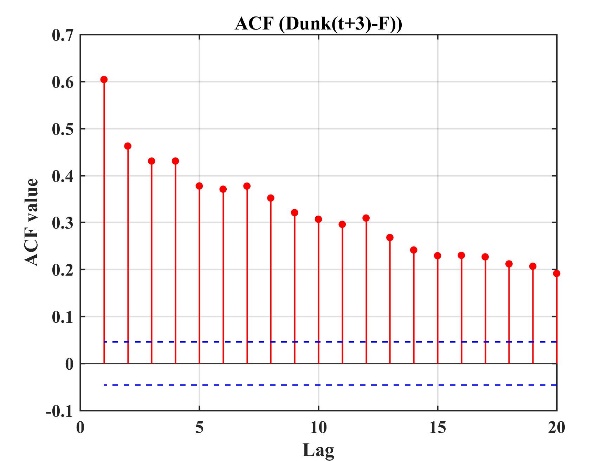 | 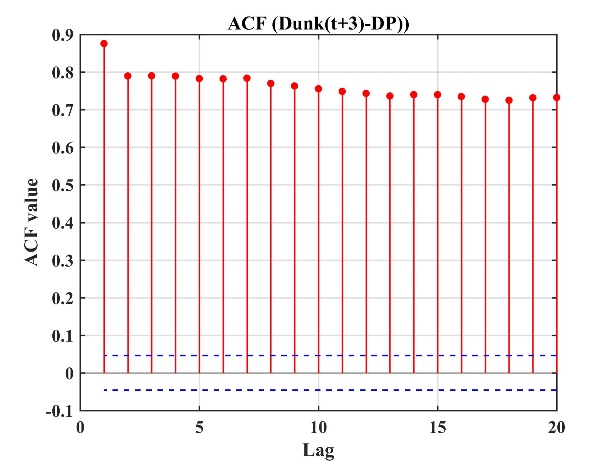 | 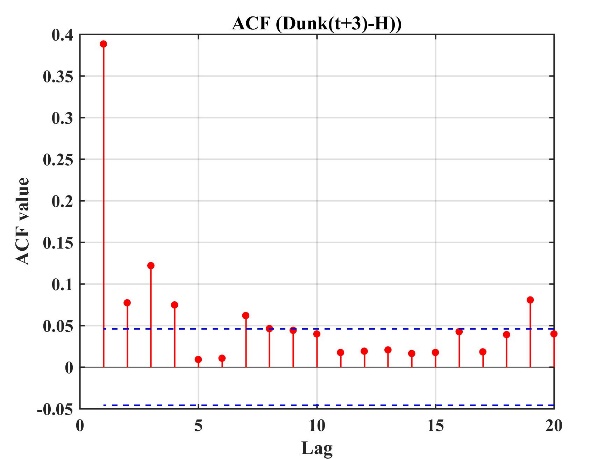 |
| **Fig. B.** ACF plot of Brook and Dunk stations | | |

**Appendix C: Scatter plots for Dunk River**

| 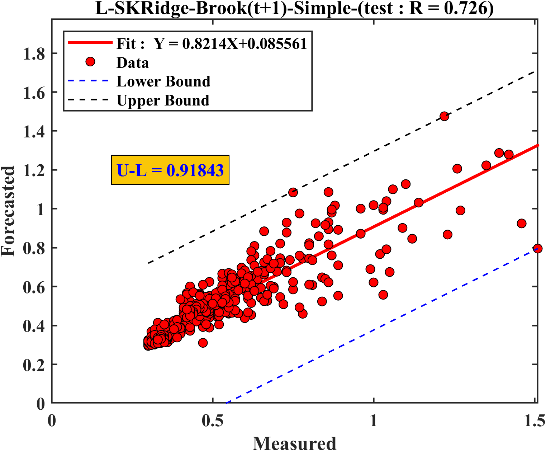 | 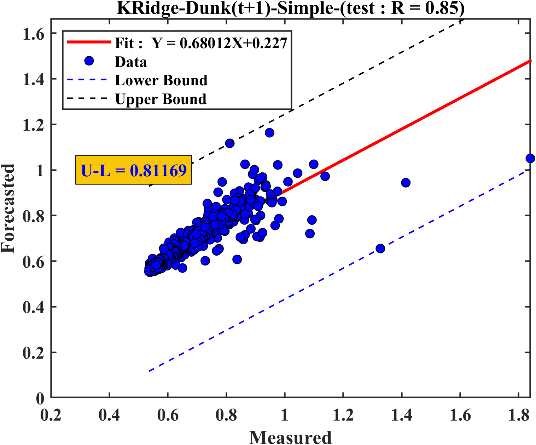 | 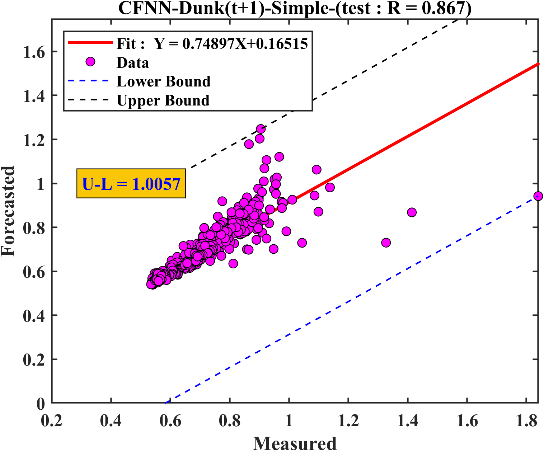 |
| --- | --- | --- |
| 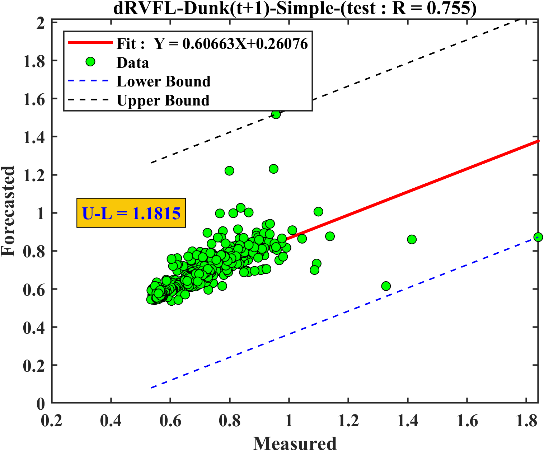 | 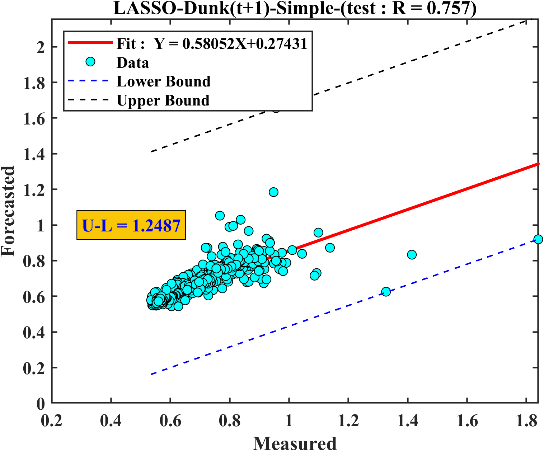 | 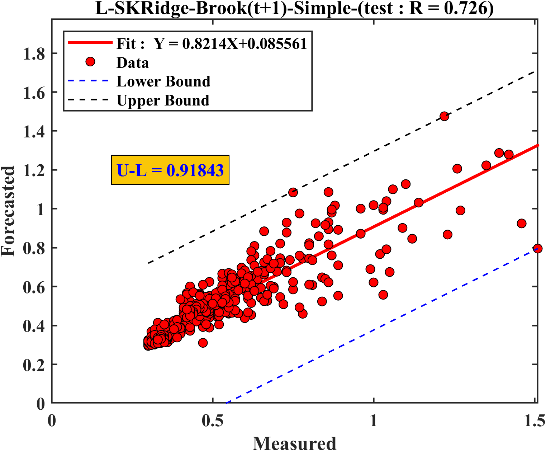 |
| 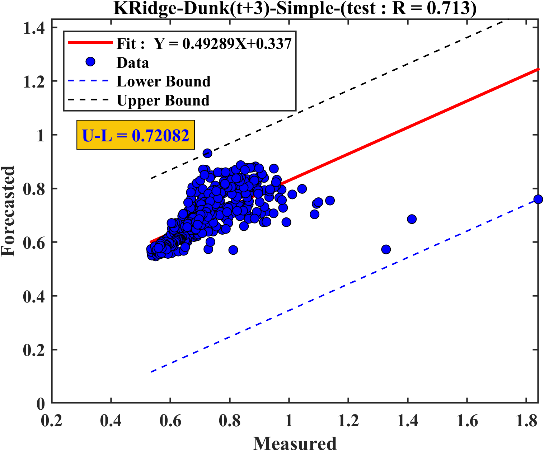 | 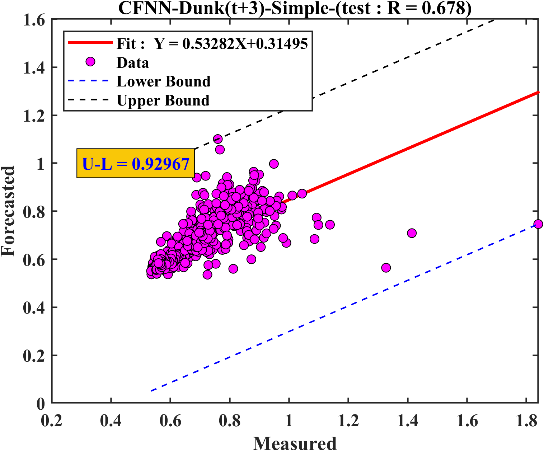 | 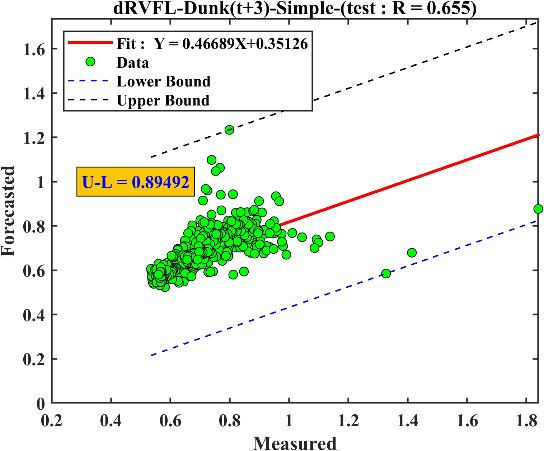 |
| 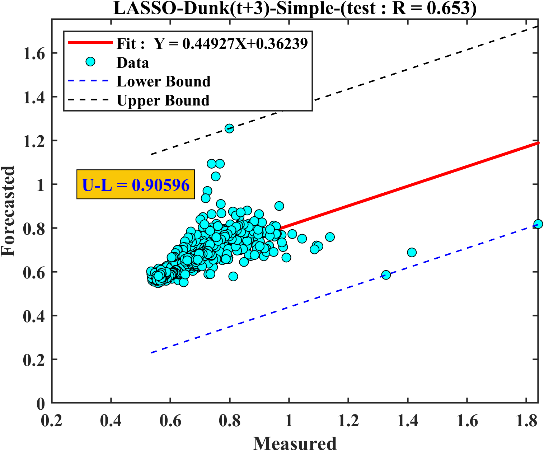 | | |
| **Fig. C1.** Scatter plots of all standalone ML models over two time horizons for Dunk river | | |

| 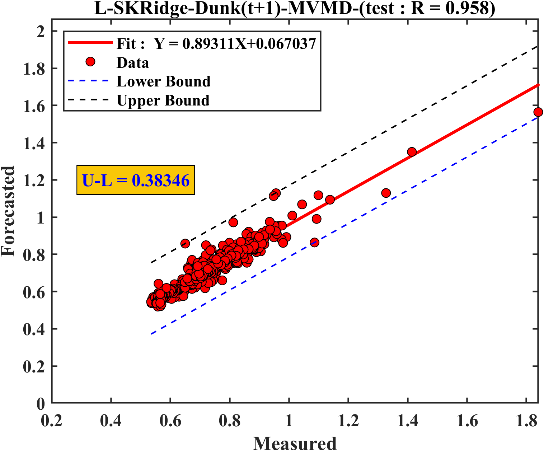 | 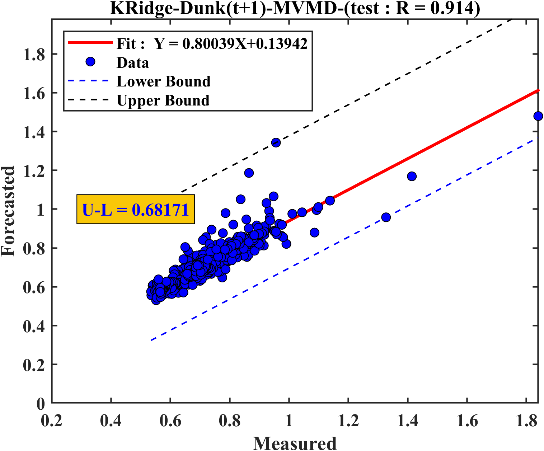 | 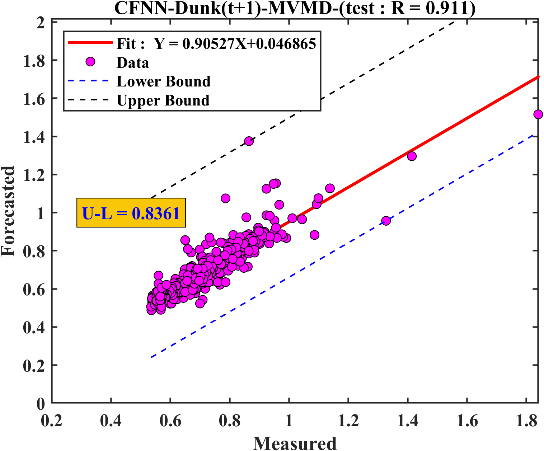 |
| --- | --- | --- |
| 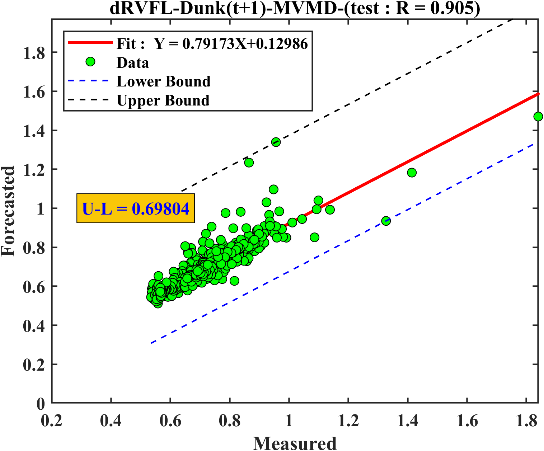 | 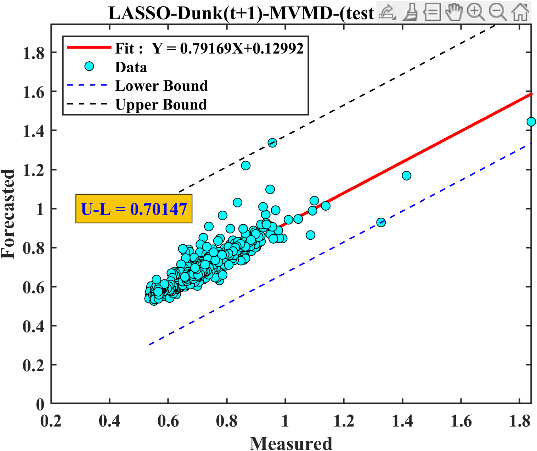 | 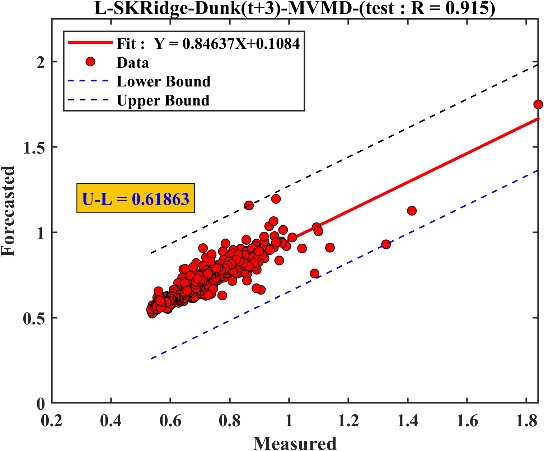 |
| 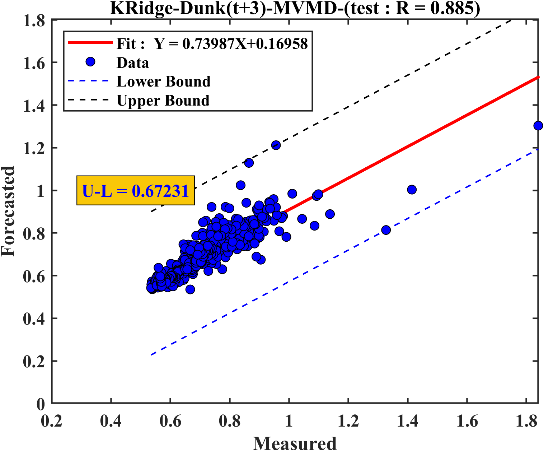 | 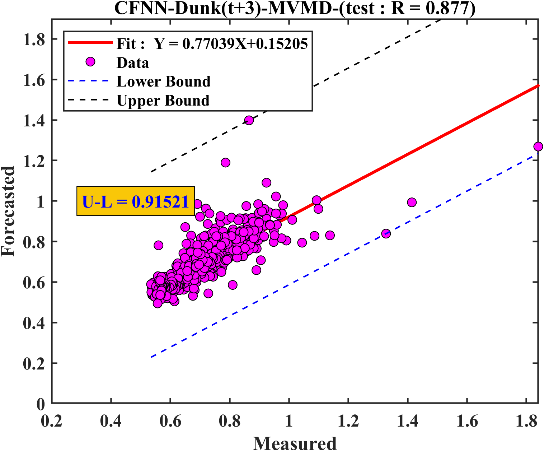 | 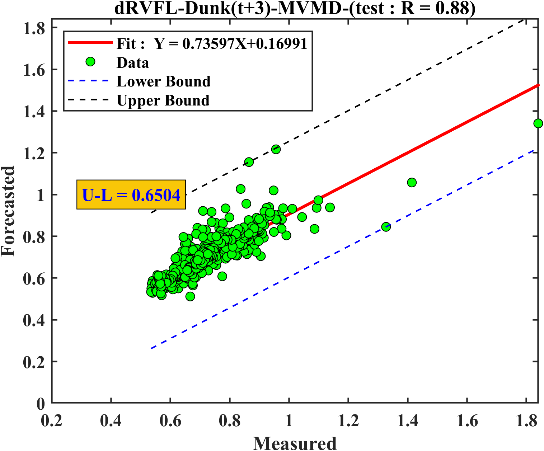 |
| 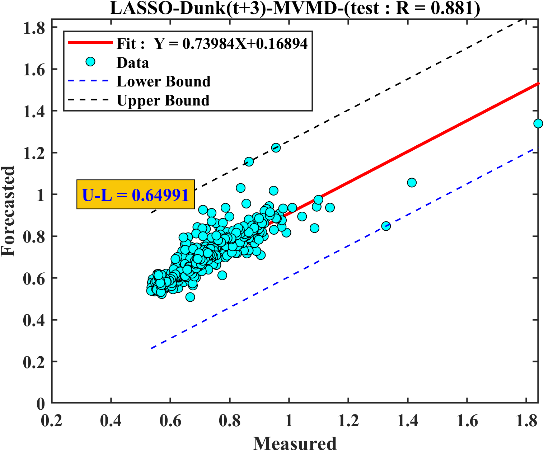 | | |
| **Fig. C2.** Scatter plots of all hybrid ML models over two time horizons for Dunk river | | |
